# Polar-Functionalized Polyethylenes Enabled by Palladium-Catalyzed Copolymerization of Ethylene and Butadiene/Bio-Based Alcohol-Derived Monomers

**DOI:** 10.3390/polym15041044

**Published:** 2023-02-19

**Authors:** Yanlin Zong, Chaoqun Wang, Yixin Zhang, Zhongbao Jian

**Affiliations:** 1 State Key Laboratory of Polymer Physics and Chemistry, Changchun Institute of Applied Chemistry, Chinese Academy of Sciences, Renmin Street 5625, Changchun 130022, China; 2 School of Applied Chemistry and Engineering, University of Science and Technology of China, Hefei 230026, China

**Keywords:** telomerization, bio-based monomers, functionalized polyethylene, palladium catalyst

## Abstract

Polar-functionalized polyolefins are high-value materials with improved properties. However, their feedstocks generally come from non-renewable fossil products; thus, it requires the development of renewable bio-based monomers to produce functionalized polyolefins. In this contribution, via the Pd-catalyzed telomerization of 1,3-butadiene and three types of bio-based alcohols (furfuryl alcohol, tetrahydrofurfuryl alcohol, and solketal), 2,7-octadienyl ether monomers including **OC8-FUR**, **OC8-THF,** and **OC8-SOL** were synthesized and characterized, respectively. The copolymerization of these monomers with ethylene catalyzed by phosphine–sulfonate palladium catalysts was further investigated. Microstructures of the resultant copolymers were analyzed by NMR and ATR-IR spectroscopy, revealing linear structures with incorporations of difunctionalized side chains bearing both allyl ether units and polar cyclic groups. Mechanical property studies exhibited better strain-at-break of these copolymers compared to the non-polar polyethylene, among which the copolymer **E-FUR** with the incorporation of 0.3 mol% displayed the highest strain-at-break and stress-at-break values of 940% and 35.9 MPa, respectively.

## 1. Introduction

Functionalized polyolefin is of great importance in both academic and industrial fields because of its significant improvement in surface properties such as wettability, adhesion, printability, and compatibility [1,2]. However, the incorporation of polar functionalities into the non-polar polyolefin backbone is highly challenging. Direct coordination–insertion copolymerization of olefin and polar monomers catalyzed by transition–metal catalysts is considered the most straightforward and powerful method to achieve this target. In this regard, late-transition–metal catalysts exhibit excellent performance on copolymerizing olefin and polar monomers, owing to their lower oxophilicity and better functional group tolerance. Over the more than the preceding two decades, various late-transition–metal catalyst systems have been developed [3,4,5,6,7,8,9], among which Drent’s type phosphine–sulfonate palladium catalysts have stood out on copolymerizing a broad scope of polar monomers, including many challenging monomers such as vinyl ether, vinyl fluoride, vinyl acetate, acrylonitrile, and acrylic acid [9,10,11,12,13,14,15,16,17,18,19,20]. In addition to the catalyst development, the design of polar monomers also enables the synthesis of functionalized polyolefin featuring distinctive chain structures [21,22,23,24,25,26,27].

Generally, feedstocks of polyolefin come from petroleum or natural gas, which are non-renewable resources. Therefore, utilizing renewable bio-based feedstocks to produce functionalized polyolefin as an alternative is of great significance from a long-term point of view [28,29,30]. Several works have successfully copolymerized bio-based monomers, such as eugenol [31,32], sugar derivatives [33], furan derivatives [34,35], and 10-undecenoic acid [36], with ethylene to afford functionalized polyethylenes. However, renewable monomers suitable for olefin copolymerization are severely limited by applicable types of bio-molecules. Thus, expanding the scope of bio-based monomers is needed, which could be achieved by converting bio-molecules to desirable monomers for olefin copolymerization.

**Scheme 1:**
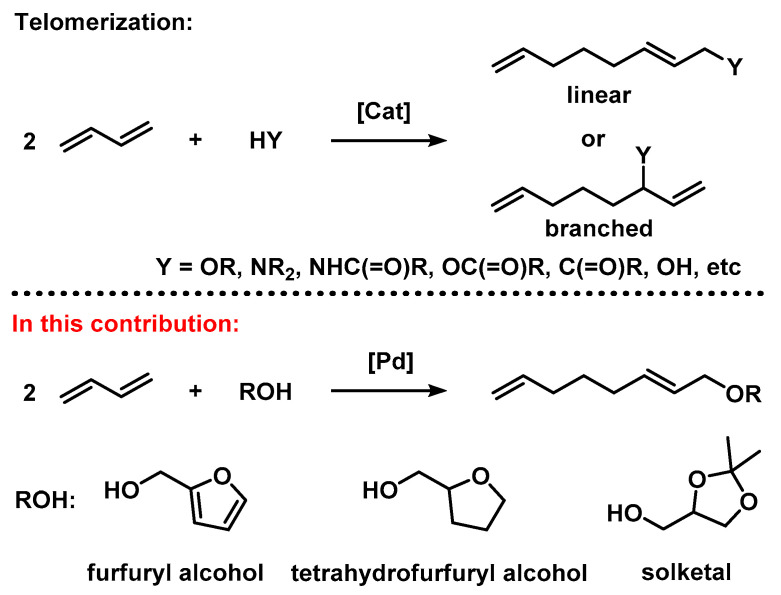
Telomerization of 1,3-butadiene with a nucleophile and synthesis of bio-derived 2,7-octadienyl ether monomers through telomerization of 1,3-butadiene and bio-based alcohols.

Telomerization is a versatile reaction that refers to a metal-catalyzed dimerization of 1,3-dienes in the presence of a nucleophile, such as alcohol, amine, amide, acid, anhydride, and water (Scheme 1) [37,38], in which the produced linear compounds can serve as potential polar olefin monomer candidates for olefin copolymerization. More importantly, this one-step reaction is not only highly active and chemo-selective but is also an environment-friendly and green chemical [39,40], providing an ideal method to produce bio-derived monomers. As shown in Scheme 1, the generated linear compounds contain two double bonds: one is a terminal double bond that can polymerize with olefin, while the other is an internal double bond that is difficult to participate in the coordination–insertion polymerization [41], but can undergo the crosslinking reaction to improve polymer properties [42]. It is worth noting that there are long spacers between the terminal double bond and the polar groups, which could decrease the possibility of poisoning the active metal center. Taking alcohol as an example, Kaminsky’s pioneering work has successfully achieved the copolymerization of ethylene with the industrial intermediate 2,7-octadienyl methyl ether that is pre-protected by alkyl aluminum [43].

Herein, we utilized the telomerization of 1,3-butadiene and bio-based alcohols to develop three 2,7-octadienyl ether monomers. Copolymerization of ethylene and these bio-derived monomers were investigated by phosphine–sulfonate palladium catalysts. The functionalities were successfully incorporated into the polyethylene backbones, and microstructures were comprehensively studied. In addition, the mechanical properties of this copolymer type were evaluated.

## 2. Materials and Methods

### 2.1. General Information

All syntheses involving air- and moisture-sensitive compounds were carried out using standard Schlenk-type glassware (or in a glove box) under an atmosphere of nitrogen. All solvents were purified from the MBraun SPS system. NMR spectra for monomers, palladium complexes, and copolymers were recorded on a Bruker AV400 (^1^H: 400 MHz, ^13^C: 100 MHz) or a Bruker AV500 (^1^H: 500 MHz, ^13^C: 125 MHz). NMR assignments were confirmed by ^1^H−^13^C HSQC and ^1^H−^13^C HMBC experiments when necessary. The molecular weights (*M*_w_) and molecular weight distributions (*M*_w_/*M*_n_) of copolymers were measured by means of gel permeation chromatography (GPC) on a PL-GPC 220-type high-temperature chromatograph equipped with three PL-gel 10 μm Mixed-B LS type columns at 150 °C. Melting temperature (*T*_m_) of copolymers was measured through DSC analyses, which were carried out on a TA Q2000 DSC Instruments under nitrogen atmosphere at heating and cooling rates of 10 °C/min (temperature range: 40–160 °C). IR spectra were acquired on a VERTEX 70 Fourier transform infrared spectrometer. Stress/strain experiments were performed at 5 mm/min on an Electromechanical Universal Testing Machine (E43.104) at room temperature. Samples were melt-pressed at 160 °C to obtain the test specimens, which have 41-mm gauge length, 17-mm width, and 1.5-mm thickness. At least two specimens of each polymer were tested.

Materials: furfuryl alcohol, tetrahydrofurfuryl alcohol, and solketal were commercially available, which were further dried over Na_2_SO_4_ and NaHCO_3_ for 2 h and distilled. Palladium catalysts **Pd-1**, **Pd-2,** and **Pd-3** were prepared using the literature procedures [44,45,46]. All other reagents were commercially available and used as received.

### 2.2. Synthesis of **Pd-3**

The synthesis of **Pd-3** was similar to **Pd-2**: Ligand was first synthesized using the procedure according to the previously reported literature [10]. The solution of ligand (300 mg, 0.68 mmol) and (tmeda)PdMe_2_ (171.8 mg, 0.68 mmol) in 1.4-dioxane (30 mL) was stirred for 24 h at room temperature. The white powder produced during the reaction was filtered and dried in a vacuum. After added to 150 mL of DMSO, the mixture was stirred at 65 °C until the white solids were all dissolved. Subsequently, the solvent was further removed to give a beige solid, which was washed 3 times with ethyl ether and dried in vacuum (350 mg, 80.8% yield) (Figures S1–S3). ^1^H NMR (500 MHz, 298 K, CDCl_3_, 7.26 ppm): δ = 8.19 (dd, 1H, aryl-*H*), 7.42 (t, 1H, aryl-*H*), 7.28 (m, 2H, aryl-*H*), 6.09 (s, 2H, aryl-*H*), 3.82 (s, 6H, OC*H*_3_), 3.78 (s, 3H, OC*H*_3_), 3.06 (s, 6H, dmso-*H*), 2.84 (m, 1H, cyclohexane-*H*), 2.32 (m, 1H, cyclohexane-*H*), 1.89 (m, 1H, cyclohexane-*H*), 1.74 (m, 1H, cyclohexane-*H*), 1.68 (m, 2H, cyclohexane-*H*), 1.49 (m, 2H, cyclohexane-*H*), 1.25 (m, 3H, cyclohexane-*H*), 0.36 (s, 3H, Pd-C*H*_3_). ^31^P NMR (202 MHz, 298 K, CDCl_3_, 7.26 ppm): δ = 23.64. ^13^C{^1^H} NMR (125 MHz, 298 K, CDCl_3_, 77.16 ppm): δ = 164.55 (*C-*OCH_3_), 162.64 (*C-*OCH_3_), 148.08 (*C*-SO_3_), 132.88 (P-*C*(PhSO_3_)), 130.24, 130.11, 129.75, 128.33, 98.29 (P-*C*(PhOMe_3_)), 91.21, 55.64 (O*C*H_3_), 55.49 (O*C*H_3_), 41.33 (S-*C*H_3_), 40.28 (P-*C*H), 32.64 (*C*H_2_), 29.75 (*C*H_2_), 27.47 (*C*H_2_), 27.31 (*C*H_2_), 26.15 (*C*H_2_),−1.03 (Pd-*C*H_3_).

### 2.3. Synthesis of 2,7-Octadienyl Ether Monomers

A general procedure: Pd(OAc)_2_ (25 mg, 0.11 mmol), 1,3-bis(2,4,6-trimethylphenyl)imidazolium chloride (IMesHCl) (151 mg, 0.44 mmol), potassium *tert*-butoxide (1.3 g, 11.58 mmol) were dissolved in the bio-based alcohol (0.22 mol). The mixture was transferred to a steel autoclave and then cooled with liquid nitrogen. After the addition of 1,3-butadiene (30 g, 0.55 mol, calculated by weight), the autoclave was sealed and heated to 90 °C. The reaction mixture was stirred for 16 h, and then excess 1,3-butadiene was removed under vacuum. The residue was distilled in vacuo, and the desired monomer was obtained as a colorless oil in the temperature range of 72 °C–80 °C in vacuo. The distilled oil was directly used for ethylene copolymerization without further purification. 

Monomer **OC8-FUR**: 70% yield. ^1^H NMR (500 MHz, CDCl_3_): δ = 7.40 (d, *J* = 1.9 Hz, 1H, furyl-*H*), 6.37–6.27 (m, 2H, furyl-*H*), 5.84–5.76 (m, 1H, CH_2_=C*H*), 5.76–5.68 (m, 1H, C*H*=CH), 5.61–5.54 (m, 1H, C*H*=CH), 5.04–4.91 (m, 2H, C*H*_2_=CH), 4.43 (s, 2H, OC*H_2_*), 3.97 (dd, *J* = 6.3, 1.2 Hz, 2H, OC*H*_2_), 2.12–2.01 (m, 4H, C*H*_2_), 1.44–1.54 (m, 2H, C*H*_2_). ^13^C NMR (125 MHz, CDCl_3_): δ = 151.89 (furyl-*C*), 142.54 (furyl-*C*), 138.41 (CH_2_=*C*H), 134.64 (*C*H=CH), 126.23 (CH=*C*H), 114.54 (*C*H_2_=CH), 110.11 (furyl-*C*), 109.00 (furyl-*C*), 70.57 (O*C*H_2_), 63.46 (O*C*H_2_), 33.11 (*C*H_2_), 31.58 (*C*H_2_), 28.14 (*C*H_2_).

Monomer **OC8-THF**: 56% yield. ^1^H NMR (500 MHz, CDCl_3_): δ = 5.80–5.71 (m, 1H, CH_2_=C*H*), 5.69–5.62 (m, 1H, C*H*=CH), 5.56–5.49 (m, 1H, C*H*=CH), 5.03–4.81 (m, 2H, C*H*_2_=CH), 4.07–3.98 (m, 1H, C*H*), 3.94 (t, *J* = 5.5 Hz, 2H, OC*H*_2_), 3.88–3.81 (m, 1H, OC*H*_2_), 3.76–3.69 (m, 1H, OC*H*_2_), 3.41–3.36 (m, 2H, OC*H*_2_), 2.06–1.99 (m, 4H, C*H*_2_), 1.96–1.88 (m, 1H, OC*H*_2_), 1.84 (dt, *J* = 14.7, 7.3 Hz, 2H, OC*H*_2_), 1.61–1.54 (m, 1H, OC*H*_2_), 1.48–1.41 (m, 2H, C*H*_2_). ^13^C NMR (125 MHz, CDCl_3_): δ = 138.60 (CH_2_=*C*H), 134.21 (*C*H=CH), 126.67 (CH=*C*H), 114.57 (*C*H_2_=CH), 77.90 (O*C*H), 72.53 (O*C*H_2_), 72.13 (O*C*H_2_), 68.26 (O*C*H_2_), 33.22 (*C*H_2_), 31.67 (*C*H_2_), 28.26 (*C*H_2_), 28.19 (*C*H_2_), 25.61 (*C*H_2_).

Monomer **OC8-SOL**: 65% yield. ^1^H NMR (500 MHz, CDCl_3_): δ = 5.81–5.72 (m, 1H, CH_2_=C*H*), 5.70–5.63 (m, 1H, C*H*=CH), 5.56–5.47 (m, 1H, C*H*=CH), 5.02–4.87 (m, 2H, C*H*_2_=CH), 4.27–4.18 (m, 1H, C*H*), 4.03 (dd, *J* = 8.2, 6.4 Hz, 1H, OC*H*_2_), 3.99–3.89 (m, 2H, OC*H*_2_), 3.69 (dd, *J* = 8.3, 6.4 Hz, 1H, OC*H*_2_), 3.48 (dd, *J* = 9.8, 5.8 Hz, 1H, OC*H*_2_), 3.39 (dd, *J* = 9.8, 5.6 Hz, 1H, OC*H*_2_), 2.09–1.99 (m, 4H, C*H*_2_), 1.48–1.40 (m, 2H, C*H*_2_), 1.40 (s, 3H, C*H*_3_), 1.33 (s, 3H, C*H*_3_). ^13^C NMR (125 MHz, CDCl_3_): δ = 138.37 (CH_2_=*C*H), 134.43 (*C*H=CH), 126.31 (CH=*C*H), 114.56 (*C*H_2_=CH), 109.20 (*C*(CH_3_)_2_), 74.66 (O*C*H), 72.11 (O*C*H_2_), 70.76 (O*C*H_2_), 66.84 (O*C*H_2_), 33.10 (*C*H_2_), 31.55 (*C*H_2_), 28.14 (*C*H_2_), 26.70 (*C*H_3_), 25.31 (*C*H_3_).

### 2.4. A General Procedure for Ethylene Copolymerization

In a typical experiment, a 150 mL glass pressure reactor connected with a high-pressure gas line was first dried at 90 °C under vacuum for at least 1 h. The reactor was then adjusted to the desired polymerization temperature. 18 mL of toluene and the desired monomer were added to the reactor under N_2_ atmosphere, and then the desired amount of the palladium catalyst in 2 mL of CH_2_Cl_2_ was injected into the polymerization system via syringe. With a rapid stirring, the reactor was pressurized and maintained at 8 bar of ethylene. After 4 h, the pressure reactor was vented, and the polymerization was quenched via the addition of 100 mL EtOH. The resulting precipitated polymers were collected and dried in a vacuum oven to a constant weight.

## 3. Results and Discussion

### 3.1. Synthesis of 2,7-Octadienyl Ether Monomers Derived from Telomerization of 1,3-Butadiene and Bio-Based Alcohols

**Scheme 2:**
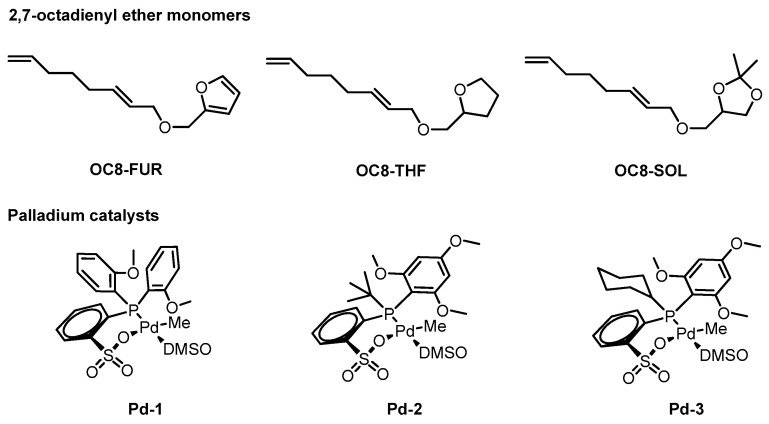
2,7-octadienyl ether monomers **OC8-FUR**, **OC8-THF,** and **OC8-SOL**, and catalysts **Pd-1** − **Pd-3** applied in copolymerization.

The Pd-catalyzed telomerization of 1,3-butadiene with a nucleophile has been extensively studied to provide a variety of diverse compounds [38,39,47,48]. Inspired by this versatile reaction, we attempted to develop a new type of 2,7-octadienyl ether monomers for ethylene copolymerization, deriving from 1,3-butadiene and bio-based alcohols. Three typical bio-based alcohols were chosen as feedstocks, among which furfuryl and tetrahydrofurfuryl alcohols are stemmed from the reduction of furfural that is industrially produced from the hydrolysis and dehydration of agricultural wastes, while solketal is derived from glycerol that is the large amount byproduct of the biodiesel production [49,50]. An in situ generated catalyst system from Pd(OAc)_2_ and the common commercial *N*-heterocyclic carbene ligand IMesHCl was used. Eventually, a series of 2,7-octadienyl ether monomers, **OC8-FUR**, **OC8-THF,** and **OC8-SOL** (Scheme 2), was successfully synthesized.

**Figure 1:**
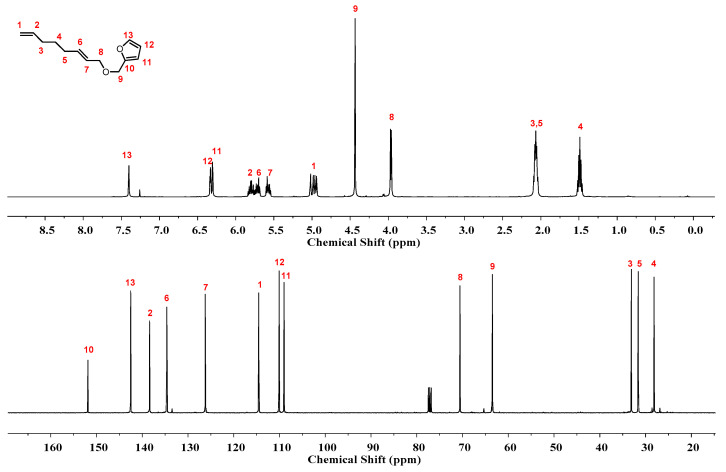
^1^H NMR and ^13^C NMR spectra of **OC8-FUR**.

These three new monomers were fully identified by 1D and 2D NMR spectroscopy (Figure S5–S14). According to ^1^H NMR analyses, the obtained 2,7-octadienyl ether featured linear structures (see details in the supporting information), indicating that these newly synthesized monomers could be good candidates for copolymerization. Taking **OC8-FUR** as an example, distinctive vinylic resonances for the terminal vinyl and the internal vinyl are clearly observed at δ = 5.79, 4.99, and 5.70, 5.59 ppm (^13^C: 138.41, 114.54, and 134.64, 126.23 ppm), respectively, in the ^1^H NMR and ^13^C NMR spectra (Figure 1). The resonances at δ = 7.40, 6.33, and 6.31 ppm were assigned to the characteristic furan ring (^13^C: 151.89, 142.54, 110.11, and 109.00 ppm).

### 3.2. Synthesis of Bio-Derived Functionalized Polyethylenes

Late-transition–metal catalysts are powerful tools to copolymerize ethylene and polar monomers due to their lower oxophilicity and higher functional group tolerance. Among these catalysts, phosphine–sulfonate palladium catalysts have been found as a universal kind of catalysts with excellent tolerance for a broad scope of polar monomers, producing highly linear functionalized polyethylene. With three selected phosphine–sulfonate palladium catalysts in hand (Scheme 2), 2,7-octadienyl ether monomers **OC8-FUR**, **OC8-THF,** and **OC8-SOL** were applied to ethylene copolymerization (Table 1).

The classical phosphine–sulfonate palladium catalyst **Pd-1** was first investigated toward ethylene copolymerization with **OC8-FUR** under different concentrations. In the presence of 0.1 M **OC8-FUR**, the highest activity of 5.35 × 10^4^ g mol^−1^ h^−1^ was achieved, along with the incorporation of 0.2 mol% (Table 1, entry 1). By increasing the concentration of **OC8-FUR** from 0.1 M to 0.3 M to 0.5 M, the incorporation increased correspondingly, reaching the highest value of 1.0 mol%, albeit with the decrease of the activity and the copolymer molecular weight, as anticipated. Under otherwise identical conditions, the activity of ethylene and 0.5 M **OC8-THF** copolymerization dropped by half compared to that of **OC8-FUR**, yet the monomer incorporation slightly increased (Table 1, entries 4 vs. 3). As for **OC8-SOL**, the highest incorporation of 1.9 mol% was observed (Table 1, entry 5), along with a higher activity than that of **OC8-THF**. It is speculated that the two methyl groups on the dioxolane ring could enhance the steric hindrance, inhibiting the coordination of the oxygen atom to the metal center. In terms of the copolymer molecular weight, the trend followed the order of **OC8-FUR** > **OC8-THF** > **OC8-SOL**.

Encouraged by the successful insertion of 2,7-octadienyl ether monomers catalyzed by **Pd-1**, two gradually bulkier palladium catalysts, **Pd-2** and **Pd-3,** were further studied (as shown in Figure S48: the steric hindrance order of these three palladium catalysts is **Pd-1** (buried volume: V_Bur_ = 45.1%) < **Pd-2** (V_Bur_ = 49.6%) < **Pd-3** (V_Bur_ = 50.8%)). For **OC8-FUR**, **Pd-2** exhibited lower activities and higher molecular weight of copolymers compared to those by **Pd-1** (Table 1, entries 6–8 vs. 1–3). These results agreed with the general rule that bulky substituents could increase the axial steric hindrance to enhance the polymer molecular weight by suppressing the β-H elimination reaction yet also slow ethylene coordination and insertion to lower activities. Moreover, the increased steric bulk could also suppress the coordination of the polar unit to the palladium center, resulting in the improvement of the monomer incorporation. Unlike those of **Pd-1**, copolymer molecular weights generated by **Pd-2** displayed an upward tendency with the monomer concentration increasing. This could be ascribed to the increasing incorporation of the monomer with a larger molecular weight than ethylene. Similar variation tends on the activity, the copolymer molecular weight and the monomer incorporation afforded by **Pd-2** were found for **OC8-THF** and **OC8-SOL** (Table 1, entries 9 vs. 4 and 10 vs. 5). Note that the highest incorporation was found for **OC8-SOL** with the value of 2.9 mol%.

**Pd-3** bearing larger steric bulky substituents further enhanced the molecular weights of **E-FUR** copolymers at monomer concentrations of 0.1 M and 0.3 M (Table 1, entries 11 and 12). However, in the presence of 0.5 M **OC8-FUR**, the copolymer molecular weight dropped dramatically (Table 1, entry 13). Compared to **Pd-2**, **Pd-3** provided lower monomer incorporations, which were similar to those of **Pd-1**. It is assumed that the steric hindrance of **Pd-3** was too large, so the incorporation of the large monomer was disfavored. As for **OC8-THF** and **OC8-SOL**, phenomena of the incorporation decrease were also observed using **Pd-3** (Table 1, entries 14 and 15). By lowering the **OC8-SOL** concentration to 0.3 M and increasing the amount of **Pd-3**, the highest molecular weight of ethylene and 2,7-octadienyl ether copolymer was generated with the value of 94.2 kDa, yet the incorporation decreased as anticipated (Table 1, entry 16). The condition of increasing the **Pd-3** amount was also applied to the copolymerization of ethylene and 0.5 M **OC8-FUR**. The monomer incorporation was improved from 1.1 mol% to 1.6 mol%, but the molecular weight slightly decreased (Table 1, entries 17 vs. 13).

### 3.3. Analysis of Copolymer Microstructures

**Figure 2:**
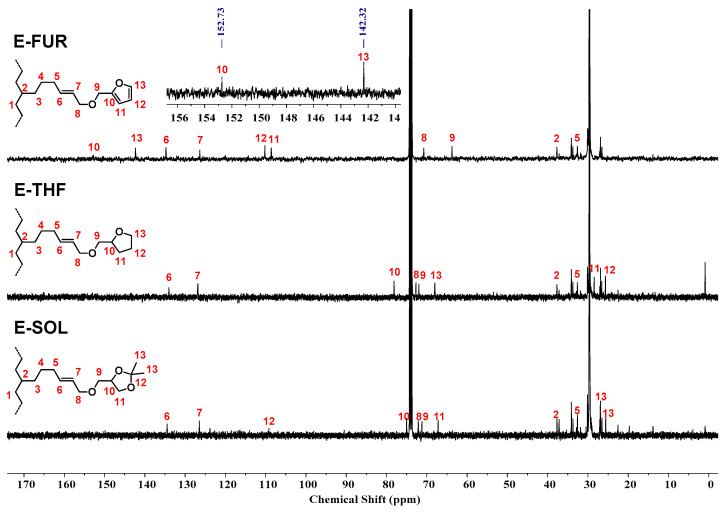
^13^C NMR spectra of ethylene and 2,7-octadienyl ether copolymers (**E-FUR**, Table 1, entry 3; **E-THF**, Table 1, entry 4; **E-SOL**, Table 1, entry 15).

Comprehensive NMR spectroscopy (Figure S15–S27), including ^1^H NMR, ^13^C NMR, ^1^H−^13^C HSQC, and ^1^H−^13^C HMBC, was further employed to identify microstructures of copolymers derived from the three 2,7-octadienyl ether monomers (see details in the supporting information). All the obtained copolymers showed linear chain structures with low branching density. As shown in Figure 2, the characteristic signals of the internal double bond appeared at δ = 134.08 and 126.49 ppm, suggesting that the ally ether unit did not participate in the coordination–insertion polymerization. This assumption was further confirmed by no observation of the cyclic structure in ^13^C NMR spectra according to previous reports on allyl ether monomers [27], which is probably because of the difficulty of the internal double bond coordination–insertion process. Additionally, key resonances of the terminal polar furfural, tetrahydrofurfuryl, and 2,2-dimethyl-1,3-dioxolane rings were clearly observed in the ^13^C NMR spectra. In a word, the ethylene and bio-based 2,7-octadienyl ether copolymers revealed a linear structure with long difunctionalized side chains, which could provide potential reaction sites for further modification.

**Figure 3:**
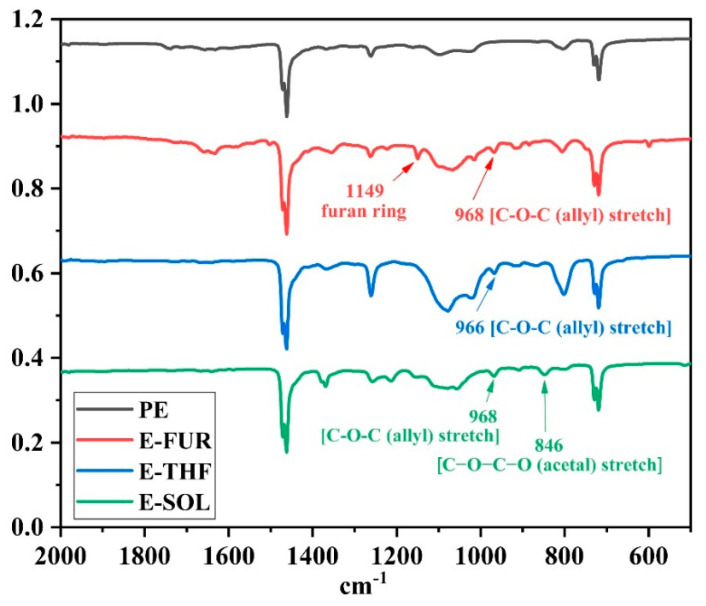
ATR-IR spectra of representative ethylene and 2,7-octadienyl ether copolymers (PE, black; **E-FUR**, red, Table 1, entry 3; **E-THF**, blue, Table 1, entry 4; **E-SOL**, green, Table 1, entry 5).

ATR-IR analyses were performed for representative ethylene and 2,7-octadienyl ether copolymers, and a polyethylene sample produced by **Pd-3** [46] was also tested as a comparison (Figure 3). As shown in Figure 3, all the copolymers feature characteristic bands at close 968 cm^−1^, assigned to the symmetric C–O–C stretching vibration of the allyl ether unit, which is absent in the PE sample. For **E-FUR**, the characteristic peak at 1148 cm^−1^ belongs to the typical absorption of furan rings [51], while the symmetric C−O−C−O stretching vibration of the acetal group is observed at 846 cm^−1^ for **E-SOL** [52]. 

### 3.4. Mechanical Properties of Copolymers

**Figure 4:**
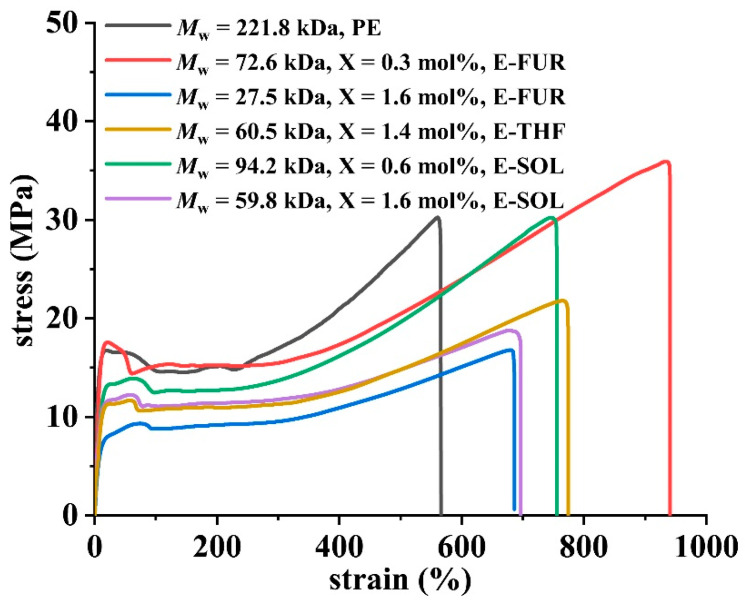
Stress–strain curves for selected functionalized polyethylene samples and a non-polar polyethylene sample generated by **Pd-3**.

Since difunctionalized polyethylenes derived from these new bio-based 2,7-octadienyl ether monomers were successfully prepared, we attempted to determine the influence of the monomer incorporation on mechanical properties. Polyethylene samples were synthesized for comparison to gain a deep understanding. However, polyethylenes produced by **Pd-1** and **Pd-2** were unable to conduct tensile tests due to low molecular weights and the brittle nature [52], which was attributed to the steric hindrance effect of the palladium catalysts on the polymer molecular weight. In principle, a lower steric hindrance in the catalyst facilitated the chain transfer of β-H elimination, which reduced the polymer molecular weight. Thus, five copolymer samples with different monomer types and monomer incorporations and a polyethylene sample afforded by **Pd-3** were selected for tensile tests to evaluate the mechanical properties (Figure 4). Typically, the branching density has a great influence on mechanical properties. Since the selected polymers generated by the phosphine–sulfonate palladium catalyst featured low branching densities, the impact of the monomer incorporation would be pronounced. As shown in Figure 4, all copolymers displayed significant improvement on strain-at-break, in contrast with the non-polar polyethylene. In view of tensile strength, copolymers with low incorporations (**E-FUR**, 0.3 mol% and **E-SOL**, 0.6 mol%) showed comparable or higher stress-at-break values compared to that of the polyethylene sample. With the increase of the monomer incorporation, the tensile strength of copolymers decreased correspondingly. It is probably because the intramolecular interactions involving the polar groups on the copolymer chain might decrease the intermolecular chain entanglement, leading to damage to the copolymer’s mechanical properties [17]. Likewise, the copolymer with lower incorporation of 0.3 mol% (vs. 1.6 mol%) had fewer branches and higher molecular weight, both of which favored tensile strength. Copolymers of **E-FUR** and **E-SOL** with the same incorporation of 1.6 mol% exhibited similar mechanical properties. In general, among the selected copolymer samples, **E-FUR** with the lowest incorporation of 0.3 mol% showed the highest values of stress-at-break and strain-at-break, which are higher than those of the compared polyethylene (35.9 MPa vs. 30.2 MPa; 940% vs. 565%). These results suggested that low incorporations of 2,7-octadienyl ether monomers are enough to be beneficial for mechanical properties.

## 4. Conclusions

In summary, three bio-derived 2,7-octadienyl ether monomers were developed through the telomerization of 1,3-butadiene and renewable bio-based alcohols. These new monomers featured linear structures with a terminal double bond, an internal double bond, and a terminal polar cyclic group. By utilizing phosphine–sulfonate palladium catalysts, polar-functionalized polyethylenes were achieved by the copolymerization of ethylene and 2,7-octadienyl ether monomers. The microstructure analyses revealed linear structures with incorporations of long side chains containing both allyl ether units and polar cyclic groups into the backbone. The copolymers with different monomer types and incorporations were tested for mechanical properties. With the incorporation of 2,7-octadienyl ether monomers, the strain-at-break values of copolymers were improved compared to the non-polar polyethylene. Furthermore, the low incorporation of polar monomers was of advantage to both stress-at-break and strain-at-break. Our work provides a potential method to expand polar monomers derived from bio-based feedstocks for olefin copolymerization to synthesize new types of functionalized polyolefins.

## Figures and Tables

**Table 1:** Copolymerization of Ethylene and Bio-derived 2,7-Octadienyl Ether Monomers ***^a^***.

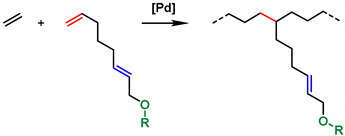										
Entry	Cat	Monomer	*c*(M) (mol L^−1^)	Yield (g)	act. ^b^ (10^4^)	*X*^c^ (mol %)	*M*_w_^d^ (10^4^)	*M*_w_*/M*_n_ ^d^	Brs ^c^	*T*_m_^e^ (°C)
**1**	**Pd-1**	**OC8-FUR**	0.1	2.14	5.35	0.2	2.20	1.86	5.6	124.3
**2**	**Pd-1**	**OC8-FUR**	0.3	0.59	1.48	0.6	2.07	1.94	4.5	120.8
**3**	**Pd-1**	**OC8-FUR**	0.5	0.67	1.68	1.0	1.60	2.21	6.0	115.1
**4**	**Pd-1**	**OC8-THF**	0.5	0.29	0.73	1.3	1.45	2.16	7.9	117.8
**5**	**Pd-1**	**OC8-SOL**	0.5	0.57	1.43	1.9	1.41	2.16	−^f^	116.5
**6**	**Pd-2**	**OC8-FUR**	0.1	0.65	1.63	0.2	4.16	1.97	4.2	123.3
**7**	**Pd-2**	**OC8-FUR**	0.3	0.21	0.53	1.4	4.40	1.89	3.3	116.0
**8**	**Pd-2**	**OC8-FUR**	0.5	0.23	0.58	2.0	4.79	2.13	7.7	114.4
**9**	**Pd-2**	**OC8-THF**	0.5	0.18	0.45	2.1	3.34	2.12	9.6	116.0
**10**	**Pd-2**	**OC8-SOL**	0.5	0.38	0.95	2.9	6.10	2.16	−^f^	117.2
**11**	**Pd-3**	**OC8-FUR**	0.1	0.79	1.98	0.3	7.26	2.18	4.4	123.9
**12**	**Pd-3**	**OC8-FUR**	0.3	0.49	1.23	0.5	7.52	1.87	0.7	118.0
**13**	**Pd-3**	**OC8-FUR**	0.5	0.18	0.45	1.1	3.66	2.13	9.1	115.4
**14**	**Pd-3**	**OC8-THF**	0.5	0.28	0.70	1.4	6.05	1.94	4.4	119.1
**15**	**Pd-3**	**OC8-SOL**	0.5	0.31	0.78	1.6	5.98	2.15	−^f^	118.1
**16 ^g^**	**Pd-3**	**OC8-SOL**	0.3	0.99	1.24	0.6	9.42	1.68	−^f^	118.3
**17 ^g^**	**Pd-3**	**OC8-FUR**	0.5	0.35	0.44	1.6	2.75	1.96	1.9	112.1

^a^ Reaction conditions: Pd catalyst (10.0 μmol), toluene/CH_2_Cl_2_ (18 mL/2 mL), 90 °C, 4 h, ethylene (8 bar), 500 rpm, at least two runs, unless otherwise noted. ^b^ Activity is in unit of g mol^−1^ h^−1 c^ *X* = Incorporation of polar monomer, Brs = Number of branches per 1000 C, as determined by ^1^H NMR in C_2_D_2_Cl_4_ at 110 °C. ^d^ Determined by GPC in 1,2,4-trichlorobenzene at 150 °C vs. linear polystyrene standards (Figure S28–S39). ^e^ Determined by DSC (second heating) (Figure S40–S47). ^f^ Not determined because it is unable to distinguish the methyl groups on the polymer chain and the 2,2-dimethyl-1,3-dioxolane. ^g^ Pd catalyst (20 μmol), toluene/CH_2_Cl_2_ (38 mL/2 mL).

## Data Availability

The data presented in this study are available in this article.

## Supplementary Materials

The following supporting information can be downloaded at: https://www.mdpi.com/article/10.3390/polym15041044/s1, Figures S1–S3: Characterization of **Pd-3**; Figures S4–S14: Characterization of 2,7-octadienyl ether monomers; Figures S15–S27: NMR figures of copolymers; Figures S28–S39: GPC figures of copolymers; Figures S40–S47: DSC figures of copolymers; Figure S48: Steric maps of **Pd-1**, **Pd-2,** and **Pd-3**; Figure S49: Possible reaction pathways in the copolymerization process.

## References

[1] Keyes A., Basbug Alhan H.E., Ordonez E., Ha U., Beezer D.B., Dau H., Liu Y.-S., Tsogtgerel E., Jones G.R., Harth E. (2019). Olefins and vinyl polar monomers: Bridging the gap for next generation materials. Angew. Chem. Int. Ed..

[2] Tan C., Zou C., Chen C. (2022). Material properties of functional polyethylenes from transition-metal-catalyzed ethylene–polar monomer copolymerization. Macromolecules.

[3] Chen Z., Brookhart M. (2018). Exploring ethylene/polar vinyl monomer copolymerizations using Ni and Pd α-diimine catalysts. Acc. Chem. Res.

[4] Mu H., Zhou G., Hu X., Jian Z. (2021). Recent advances in nickel mediated copolymerization of olefin with polar monomers. Coord. Chem. Rev..

[5] Guo L., Dai S., Sui X., Chen C. (2016). Palladium and nickel catalyzed chain walking olefin polymerization and copolymerization. ACS Catal..

[6] Mu H., Pan L., Song D., Li Y. (2015). Neutral nickel catalysts for olefin homo- and copolymerization: Relationships between catalyst structures and catalytic properties. Chem. Rev..

[7] Pasini D., Takeuchi D. (2018). Cyclopolymerizations: Synthetic tools for the precision synthesis of macromolecular architectures. Chem. Rev..

[8] Luckham S.L.J., Nozaki K. (2021). Toward the copolymerization of propylene with polar comonomers. Acc. Chem. Res..

[9] Zhang Y., Zhang Y., Hu X., Wang C., Jian Z. (2022). Advances on controlled chain walking and suppression of chain transfer in catalytic olefin polymerization. ACS Catal..

[10] Xia J., Han Y.-F., Kou S., Zhang Y., Jian Z. (2021). Exploring steric effect of electron-donating group in palladium and nickel mediated ethylene polymerization and copolymerization with polar monomers. Eur. Polym. J..

[11] Zhang Y., Jian Z. (2020). Comprehensive picture of functionalized vinyl monomers in chain-walking polymerization. Macromolecules.

[12] Wu Z., Hong C., Du H., Pang W., Chen C. (2017). Influence of ligand backbone structure and connectivity on the properties of phosphine-sulfonate Pd(II)/Ni(II) catalysts. Polymers.

[13] Xia J., Zhang Y., Hu X., Ma X., Cui L., Zhang J., Jian Z. (2019). Sterically very bulky aliphatic/aromatic phosphine-sulfonate palladium catalysts for ethylene polymerization and copolymerization with polar monomers. Polym. Chem..

[14] Wang X., Nozaki K. (2018). Selective chain-end functionalization of polar polyethylenes: Orthogonal reactivity of carbene and polar vinyl monomers in their copolymerization with ethylene. J. Am. Chem. Soc..

[15] Zhang D., Chen C. (2017). Influence of polyethylene glycol unit on palladium- and nickel-catalyzed ethylene polymerization and copolymerization. Angew. Chem. Int. Ed..

[16] Wada S., Jordan R.F. (2017). Olefin insertion into a Pd-F bond: Catalyst reactivation following β-F elimination in ethylene/vinyl fluoride copolymerization. Angew. Chem. Int. Ed..

[17] Na Y., Dai S., Chen C. (2018). Direct synthesis of polar-functionalized linear low-density polyethylene (LLDPE) and low-density polyethylene (LDPE). Macromolecules.

[18] Gaikwad S.R., Deshmukh S.S., Koshti V.S., Poddar S., Gonnade R.G., Rajamohanan P.R., Chikkali S.H. (2017). Reactivity of difunctional polar monomers and ethylene copolymerization: A comprehensive account. Macromolecules.

[19] Jian Z., Baier M.C., Mecking S. (2015). Suppression of chain transfer in catalytic acrylate polymerization via rapid and selective secondary insertion. J. Am. Chem. Soc..

[20] Ota Y., Ito S., Kuroda J., Okumura Y., Nozaki K. (2014). Quantification of the steric influence of alkylphosphine–sulfonate ligands on polymerization, leading to high-molecular-weight copolymers of ethylene and polar monomers. J. Am. Chem. Soc..

[21] Zhou G., Cui L., Mu H., Jian Z. (2021). Custom-made polar monomers utilized in nickel and palladium promoted olefin copolymerization. Polym. Chem..

[22] Zhang Y., Xia J., Song J., Zhang J., Ni X., Jian Z. (2019). Combination of ethylene, 1,3-butadiene, and carbon dioxide into ester-functionalized polyethylenes via palladium-catalyzed coupling and insertion polymerization. Macromolecules.

[23] Tang S., Zhao Y., Nozaki K. (2021). Accessing divergent main-chain-functionalized polyethylenes via copolymerization of ethylene with a CO_2_/butadiene-derived lactone. J. Am. Chem. Soc..

[24] Wang X., Seidel F.W., Nozaki K. (2019). Synthesis of polyethylene with in-chain α, β-unsaturated ketone and isolated ketone units: Pd-catalyzed ring-opening copolymerization of cyclopropenone with ethylene. Angew. Chem. Int. Ed..

[25] Cui L., Chen M., Chen C., Liu D., Jian Z. (2019). Systematic studies on (co)polymerization of polar styrene monomers with palladium catalysts. Macromolecules.

[26] Jian Z., Mecking S. (2016). Insertion polymerization of divinyl formal. Macromolecules.

[27] Jian Z., Mecking S. (2015). Insertion homo- and copolymerization of diallyl ether. Angew. Chem. Int. Ed..

[28] Fagnani D.E., Tami J.L., Copley G., Clemons M.N., Getzler Y.D.Y.L., McNeil A.J. (2021). 100th anniversary of macromolecular science viewpoint: Redefining sustainable polymers. ACS Macro Lett..

[29] Schneiderman D.K., Hillmyer M.A. (2017). 50th anniversary perspective: There is a great future in sustainable polymers. Macromolecules.

[30] Zhu Y., Romain C., Williams C.K. (2016). Sustainable polymers from renewable resources. Nature.

[31] Na Y., Chen C. (2020). Catechol-functionalized polyolefins. Angew. Chem. Int. Ed..

[32] Parisi L.R., Scheibel D.M., Lin S., Bennett E.M., Lodge J.M., Miri M.J. (2017). Eugenol as renewable comonomer compared to 4-penten-1-ol in ethylene copolymerization using a palladium aryl sulfonate catalyst. Polymer.

[33] Rajput B.S., Pawal S.B., Bodkhe D.V., Rao I.N., Sainath A.V.S., Chikkali S.H. (2020). Renewing polyethylene: Insertion copolymerization of sugar derived hydrophilic monomers with ethylene. Eur. Polym. J..

[34] Du C., Zhong L., Gao J., Zhong S., Liao H., Gao H., Wu Q. (2019). Living (co)polymerization of ethylene and bio-based furfuryl acrylate using dibenzobarrelene derived α-diimine palladium catalysts. Polym. Chem..

[35] Jian Z., Falivene L., Boffa G., Sánchez S.O., Caporaso L., Grassi A., Mecking S. (2016). Direct synthesis of telechelic polyethylene by selective insertion polymerization. Angew. Chem. Int. Ed..

[36] Dai S., Li S., Xu G., Chen C. (2020). Direct synthesis of polar functionalized polyethylene thermoplastic elastomer. Macromolecules.

[37] Behr A., Becker M., Beckmann T., Johnen L., Leschinski J., Reyer S. (2009). Telomerization: Advances and applications of a versatile reaction. Angew. Chem. Int. Ed..

[38] Clement N.D., Routaboul L., Grotevendt A., Jackstell R., Beller M. (2008). Development of palladium–carbene catalysts for telomerization and dimerization of 1,3-dienes: From basic research to industrial applications. Chem. Eur. J..

[39] Jackstell R., Harkal S., Jiao H., Spannenberg A., Borgmann C., Röttger D., Nierlich F., Elliot M., Niven S., Cavell K. (2004). An industrially viable catalyst system for palladium-catalyzed telomerizations of 1,3-butadiene with alcohols. Chem. Eur. J..

[40] Jackstell R., Andreu G.M., Frisch A., Selvakumar K., Zapf A., Klein H., Spannenberg A., Röttger D., Briel O., Karch R. (2002). A highly efficient catalyst for the telomerization of 1,3-dienes with alcohols: First synthesis of a monocarbenepalladium(0)-olefin complex. Angew. Chem. Int. Ed..

[41] Chen M., Chen C. (2020). Direct and tandem routes for the copolymerization of ethylene with polar functionalized internal olefins. Angew. Chem. Int. Ed..

[42] Smedberg A., Hjertberg T., Gustafsson B. (1997). Crosslinking reactions in an unsaturated low density polyethylene. Polymer.

[43] Fernandes M., Kaminsky W. (2009). Copolymerization of ethylene with 2,7-octadienyl methyl ether in the presence of metallocene and nickel diimine catalysts. Macromol. Chem. Phys..

[44] Guironnet D., Roesle P., Rünzi T., Göttker-Schnetmann I., Mecking S. (2009). Insertion polymerization of acrylate. J. Am. Chem. Soc..

[45] Wucher P., Goldbach V., Mecking S. (2013). Electronic influences in phosphinesulfonato palladium(II) polymerization catalysts. Organometallics.

[46] Wang C., Xia J., Zhang Y., Hu X., Jian Z. (2023). Photodegradable polar functionalized polyethylenes. Natl. Sci. Rev..

[47] Vogelsang D., Vondran J., Vorholt A.J. (2018). One-step palladium catalysed synthetic route to unsaturated pelargonic C_9_-amides directly from 1,3-butadiene. J. Catal..

[48] Behr A., Beckmann T., Schwach P. (2008). Multiphase telomerisation of butadiene with acetic acid and acetic anhydride. J. Organomet. Chem..

[49] Besson M., Gallezot P., Pinel C. (2014). Conversion of biomass into chemicals over metal catalysts. Chem. Rev..

[50] Lanzafame P., Centi G., Perathoner S. (2014). Catalysis for biomass and CO_2_ use through solar energy: Opening new scenarios for a sustainable and low-carbon chemical production. Chem. Soc. Rev..

[51] Zhang Y., Broekhuis A.A., Picchioni F. (2009). Thermally self-healing polymeric materials: The next step to recycling thermoset polymers?. Macromolecules.

[52] Odenwald L., Wimmer F.P., Mast N.K., Schußmann M.G., Wilhelm M., Mecking S. (2022). Molecularly defined polyolefin vitrimers from catalytic insertion polymerization. J. Am. Chem. Soc..

