# Supplementary material for: Polar-Functionalized Polyethylenes Enabled by Palladium-Catalyzed Copolymerization of Ethylene and Butadiene/Bio-Based Alcohol-Derived Monomers
Source: Polymers (Basel). 2023 Feb 19;15(4):1044. doi: 10.3390/polym15041044 (PMC9967981; doi:10.3390/polym15041044)
Supplement: Supplementary file 1 [file polymers-15-01044-s001.zip › polymers-2162302-supplementary.pdf]

Supplementary Information

Polar Functionalized Polyethylenes Enabled by Palladium Catalyzed Copolymerization of Ethylene and Butadiene/Bio-Based Alcohol Derived Monomers

Yanlin Zong,[1,2] Chaoqun Wang[1,2], Yixin Zhang[1,*] and Zhongbao Jian[1,2,*]

[1] State Key Laboratory of Polymer Physics and Chemistry, Changchun Institute of Applied Chemistry, Chinese Academy of Sciences, Renmin Street 5625, Changchun 130022, China
[2] University of Science and Technology of China, Hefei 230026, China

Contents

1. Characterization of Pd-3 2

2. Characterization of 2,7-octadienyl ether monomers 3

3. NMR figures of copolymers 9

4. GPC figures of copolymers 16

5. DSC figures of copolymers 20

6. Steric maps of Pd-1, Pd-2 and Pd-3 24

7. Possible reaction pathways in the copolymerization process 24

1. Characterization of Pd-3

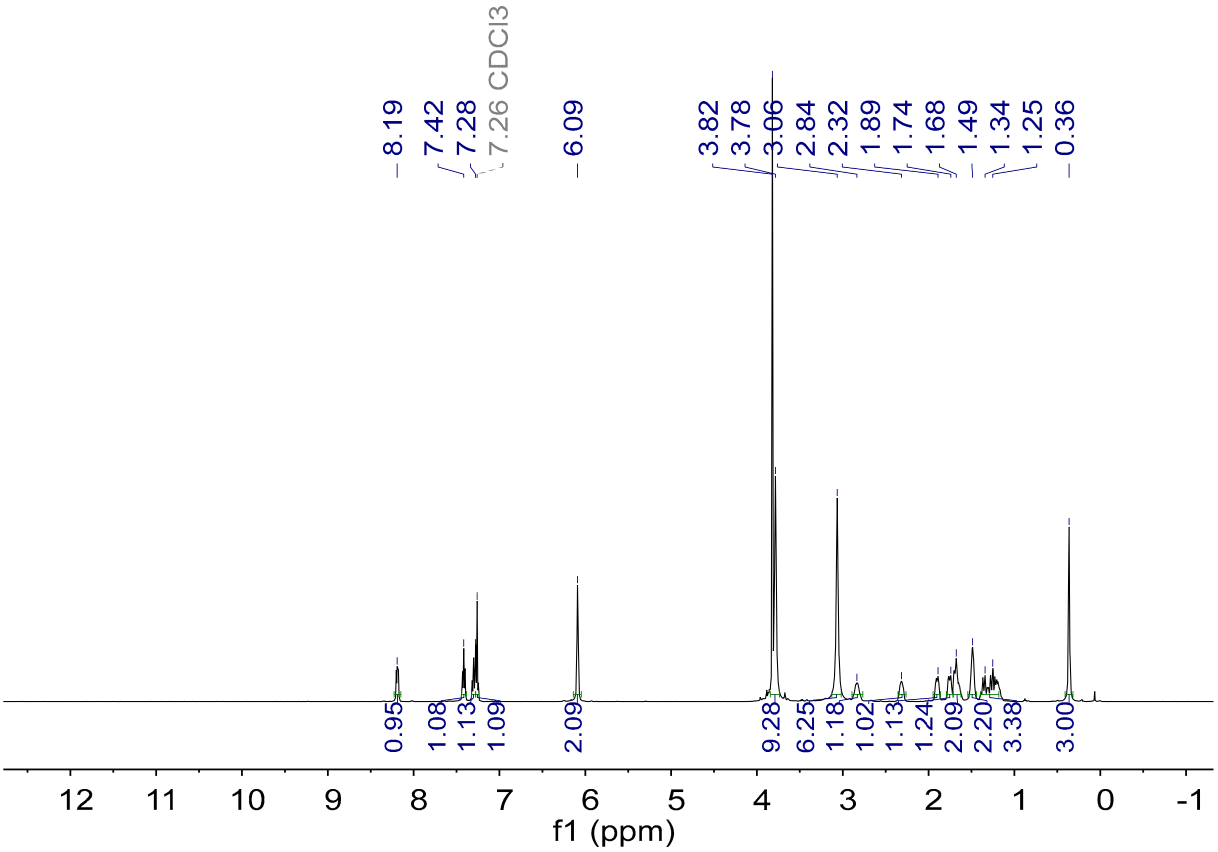

Figure S1. ^{1}H NMR spectrum of **Pd-3** in $CDCl_3$.

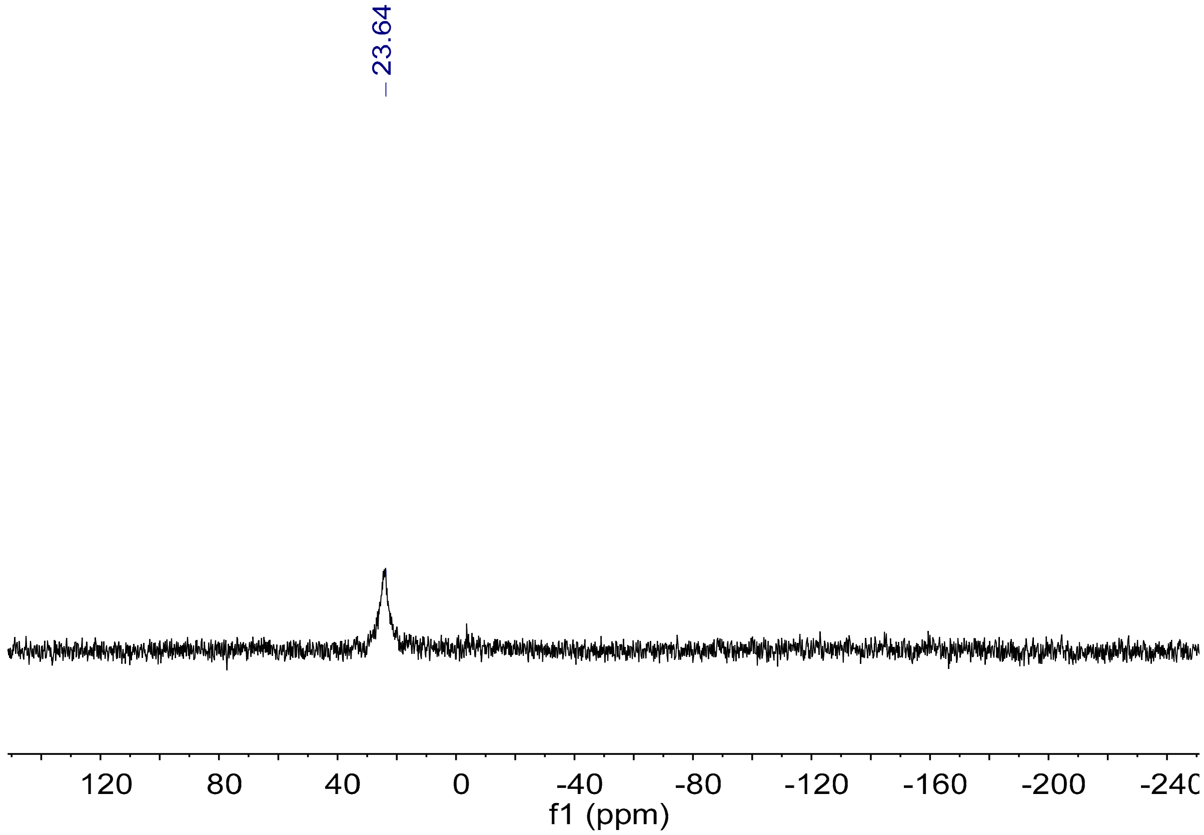

Figure S2. ^{31}P NMR spectrum of **Pd-3** in $CDCl_3$.

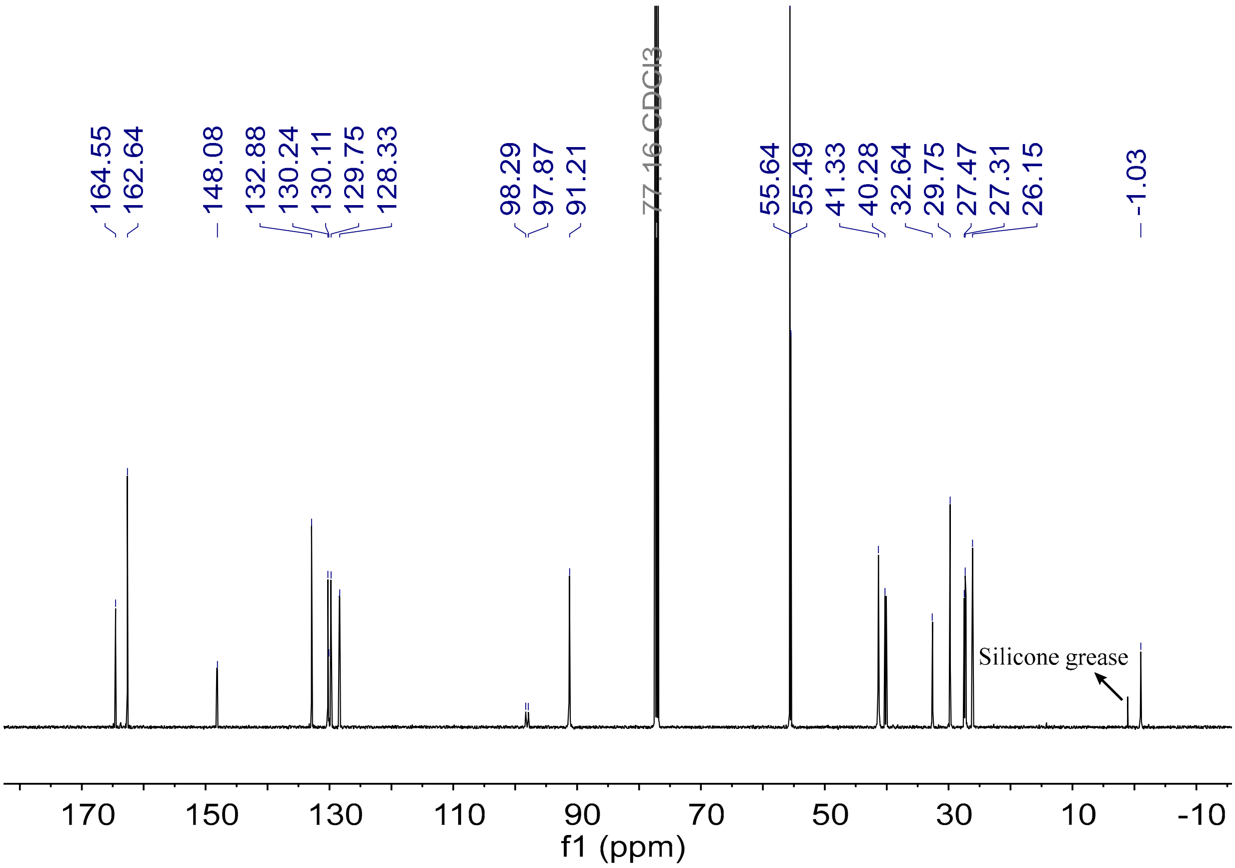

Figure S3. ^{13}C NMR spectrum of **Pd-3** in $CDCl_3$.

2. Characterization of 2,7-octadienyl ether monomers

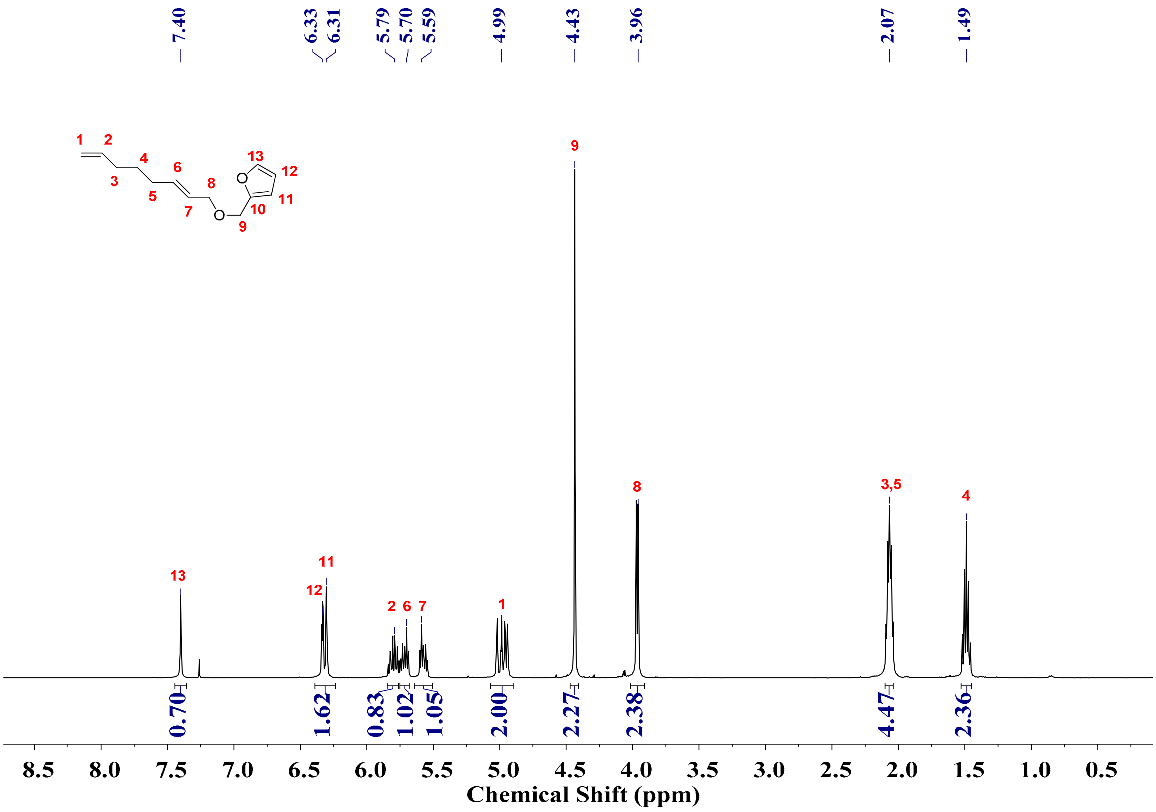

Figure S4. ^{1}H NMR spectrum of **OC8-FUR** in $CDCl_3$.

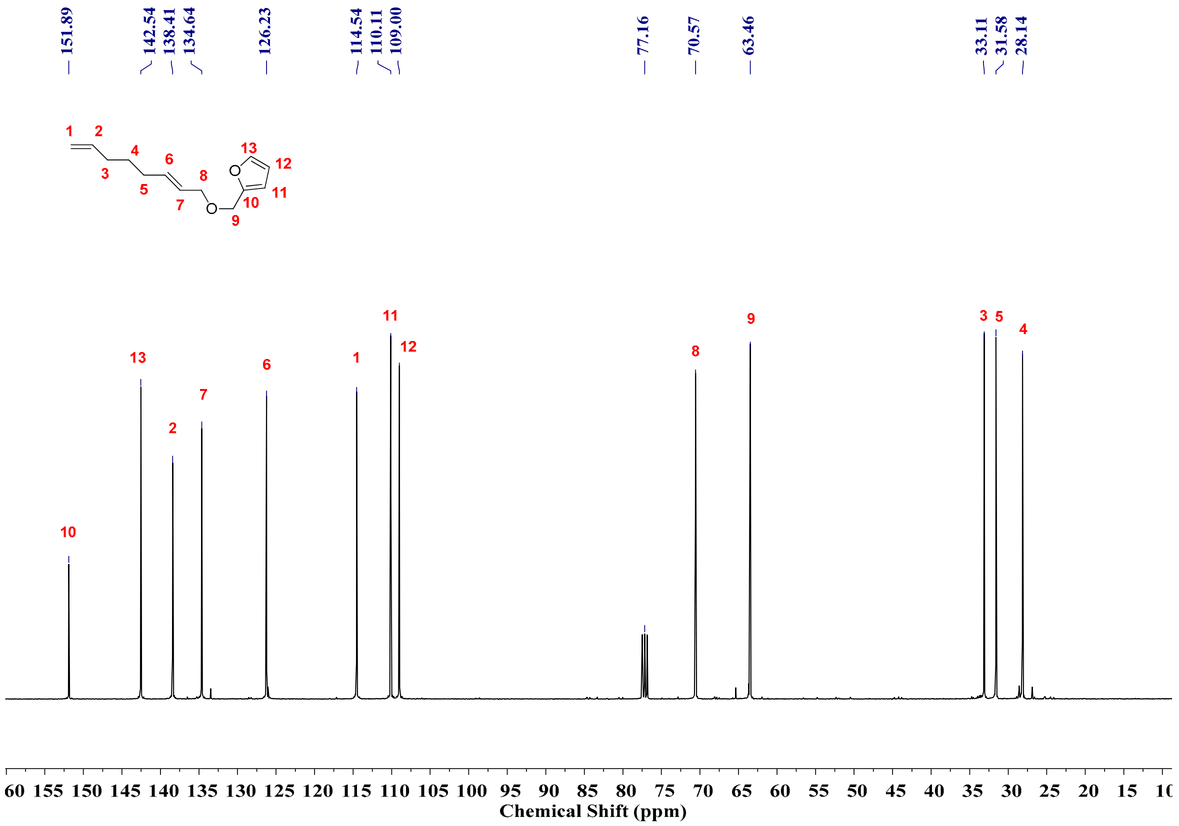

Figure S5. ^{13}C NMR spectrum of **OC8-FUR** in $CDCl_3$.

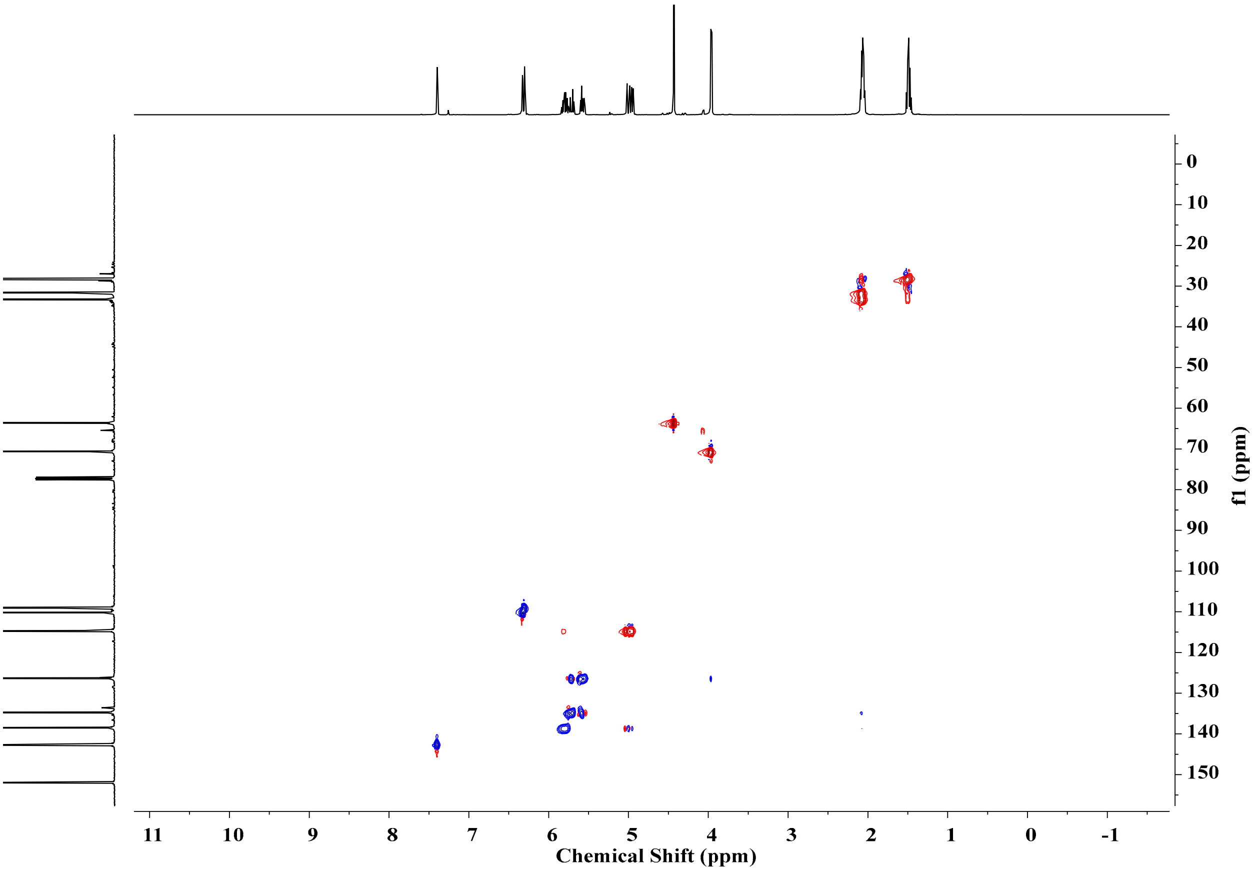

Figure S6. ^{1}H-^{13}C HSQC spectrum of **OC8-FUR** in $CDCl_3$.

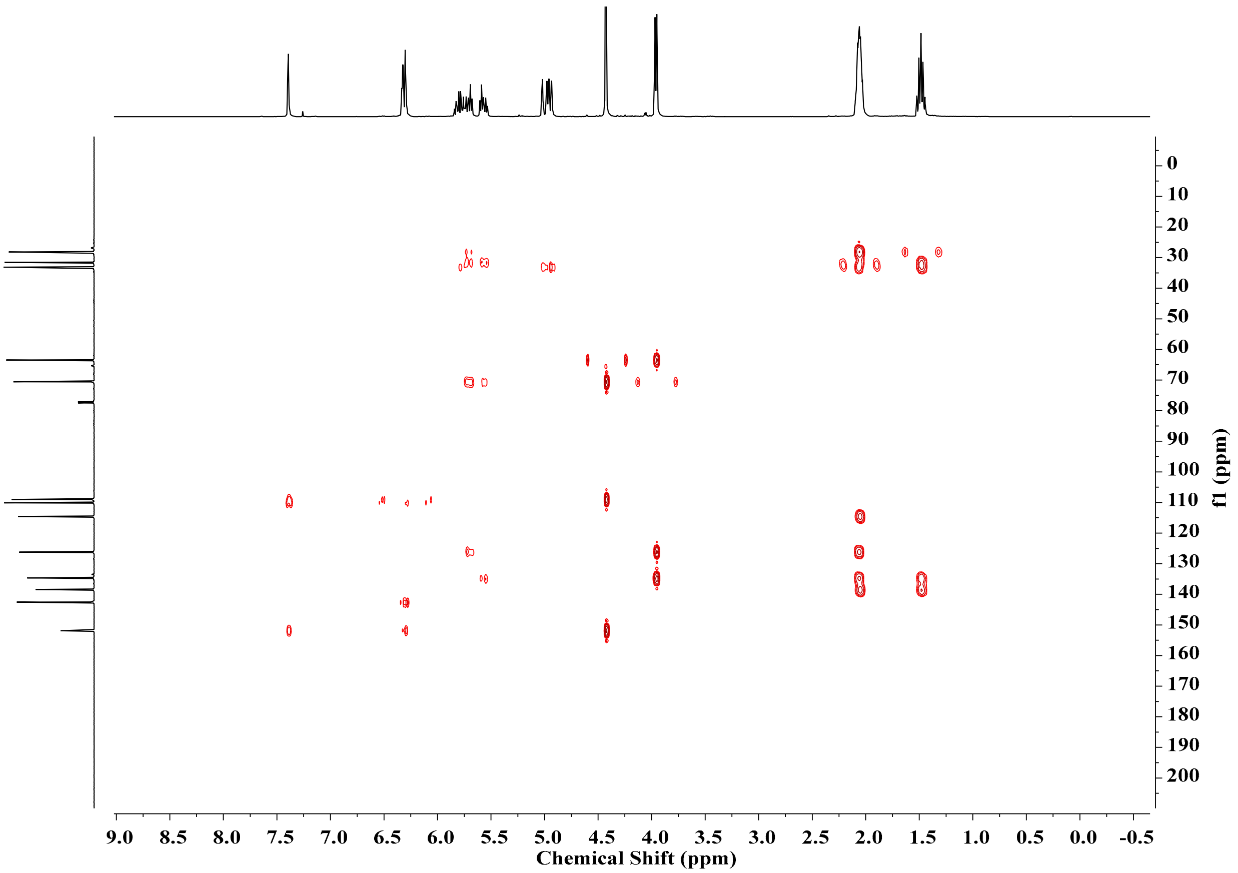

Figure S7. ^{1}H-^{13}C HMBC spectrum of **OC8-FUR** in $CDCl_3$.

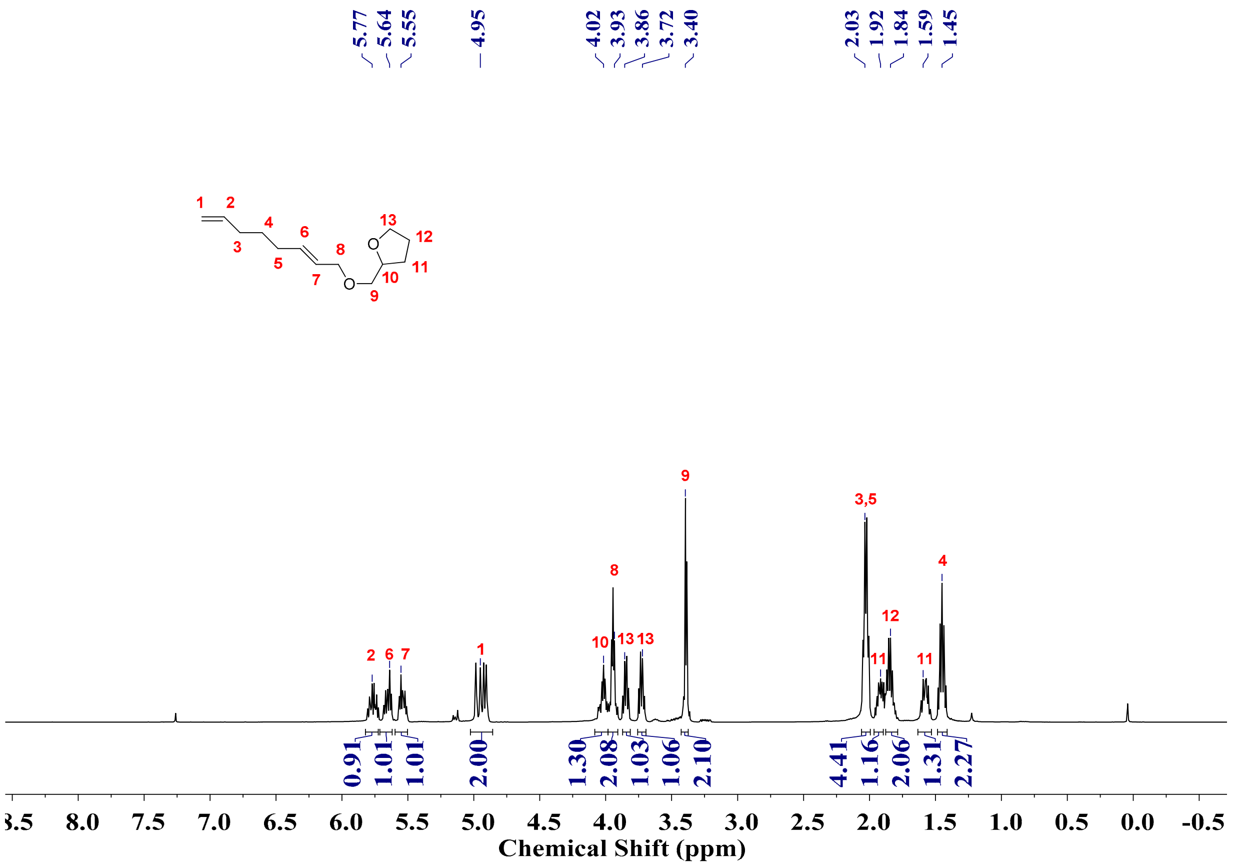

Figure S8. ^{1}H NMR spectrum of **OC8-THF** in $CDCl_3$.

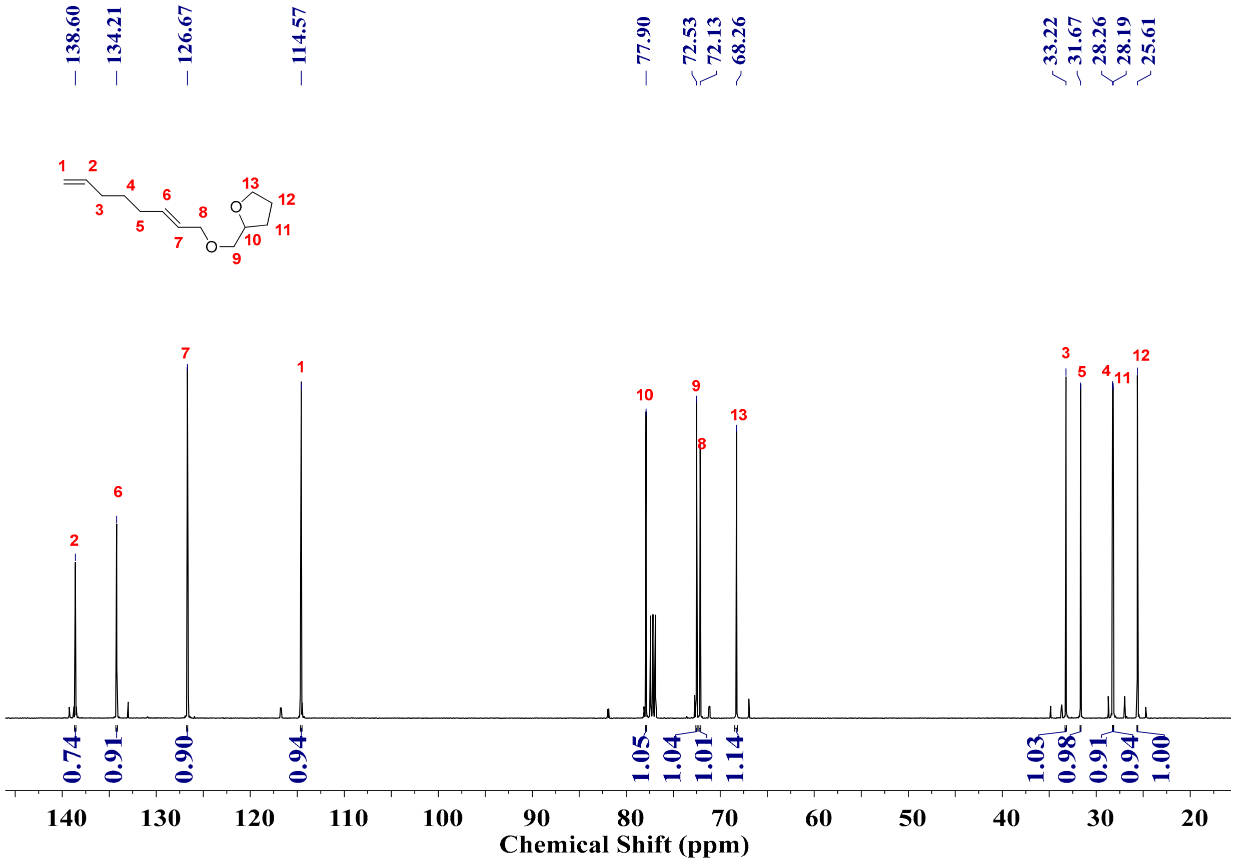

Figure S9. ^{13}C NMR spectrum of **OC8-THF** in $CDCl_3$.

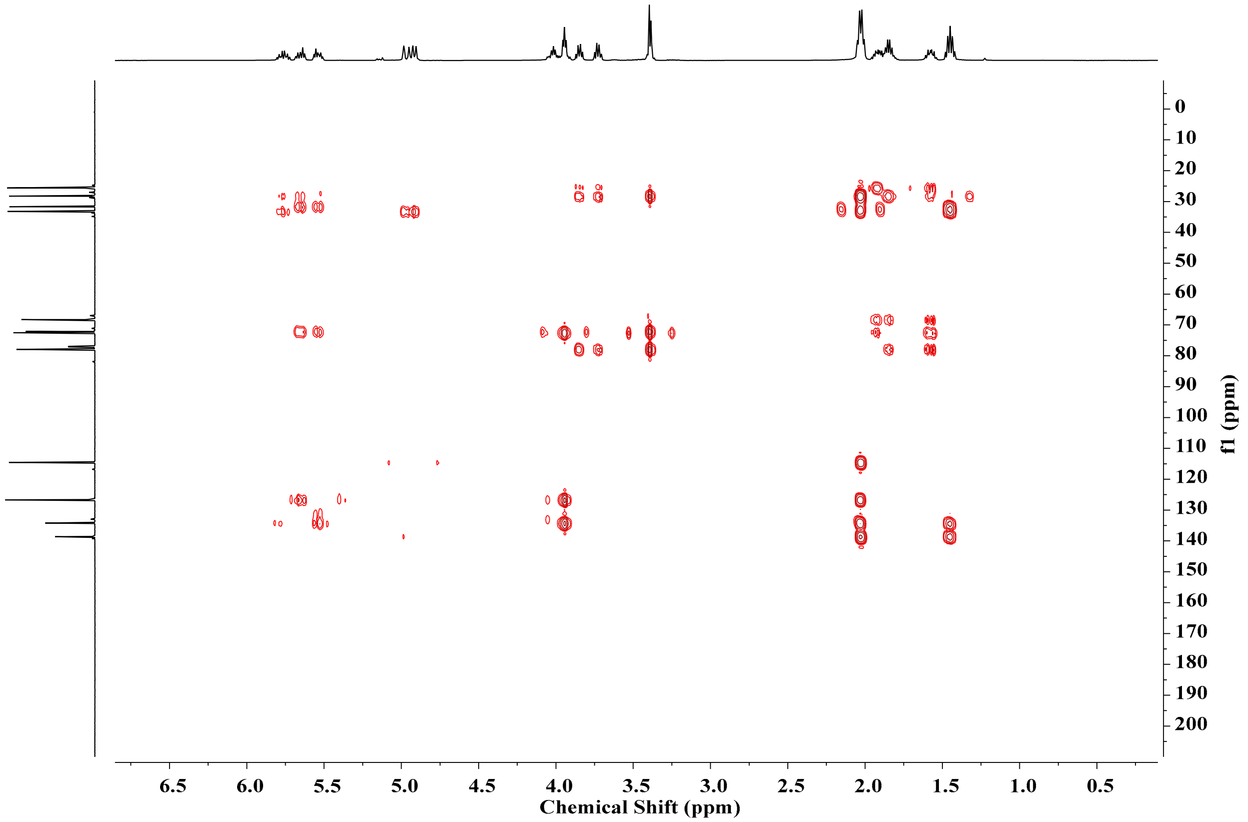

Figure S10. ^{1}H-^{13}C HMBC spectrum of **OC8-THF** in $CDCl_3$.

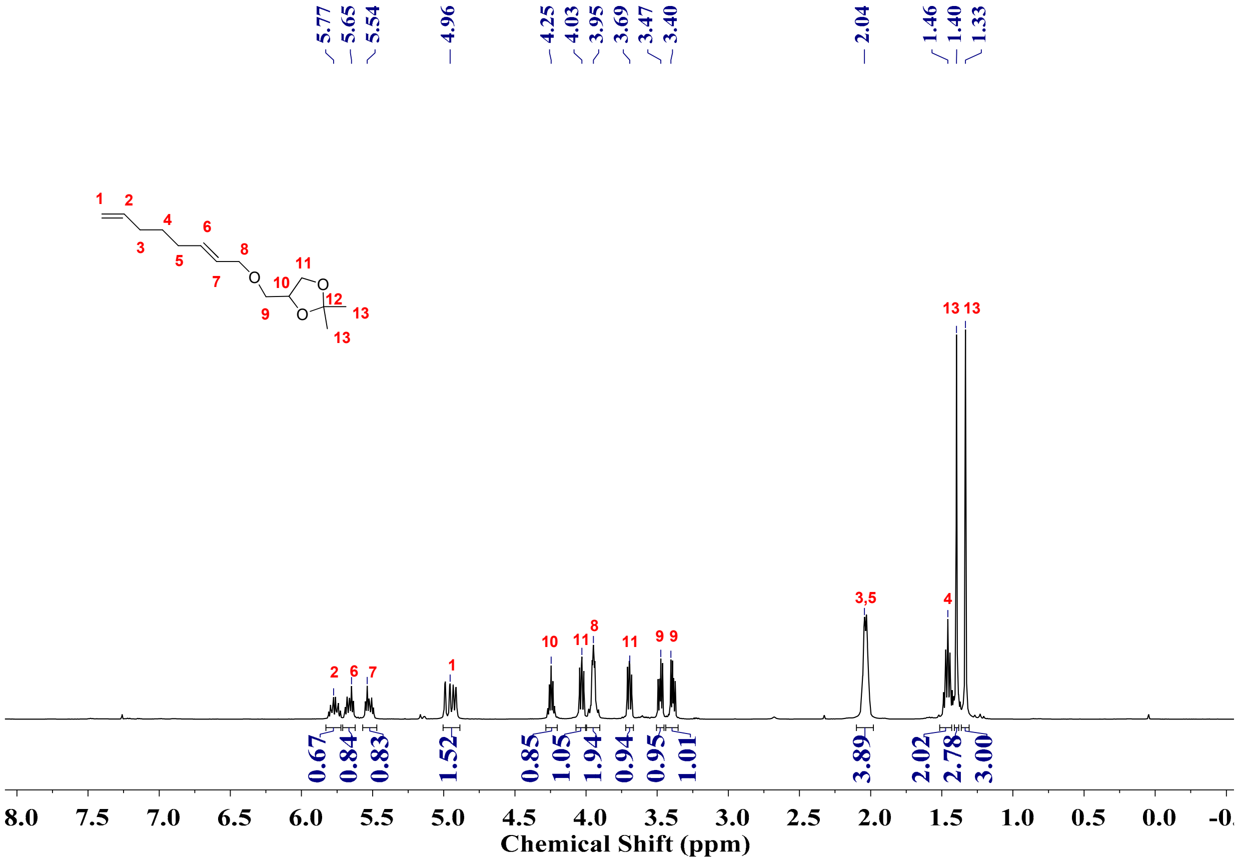

Figure S11. ^{1}H NMR spectrum of **OC8-SOL** in $CDCl_3$.

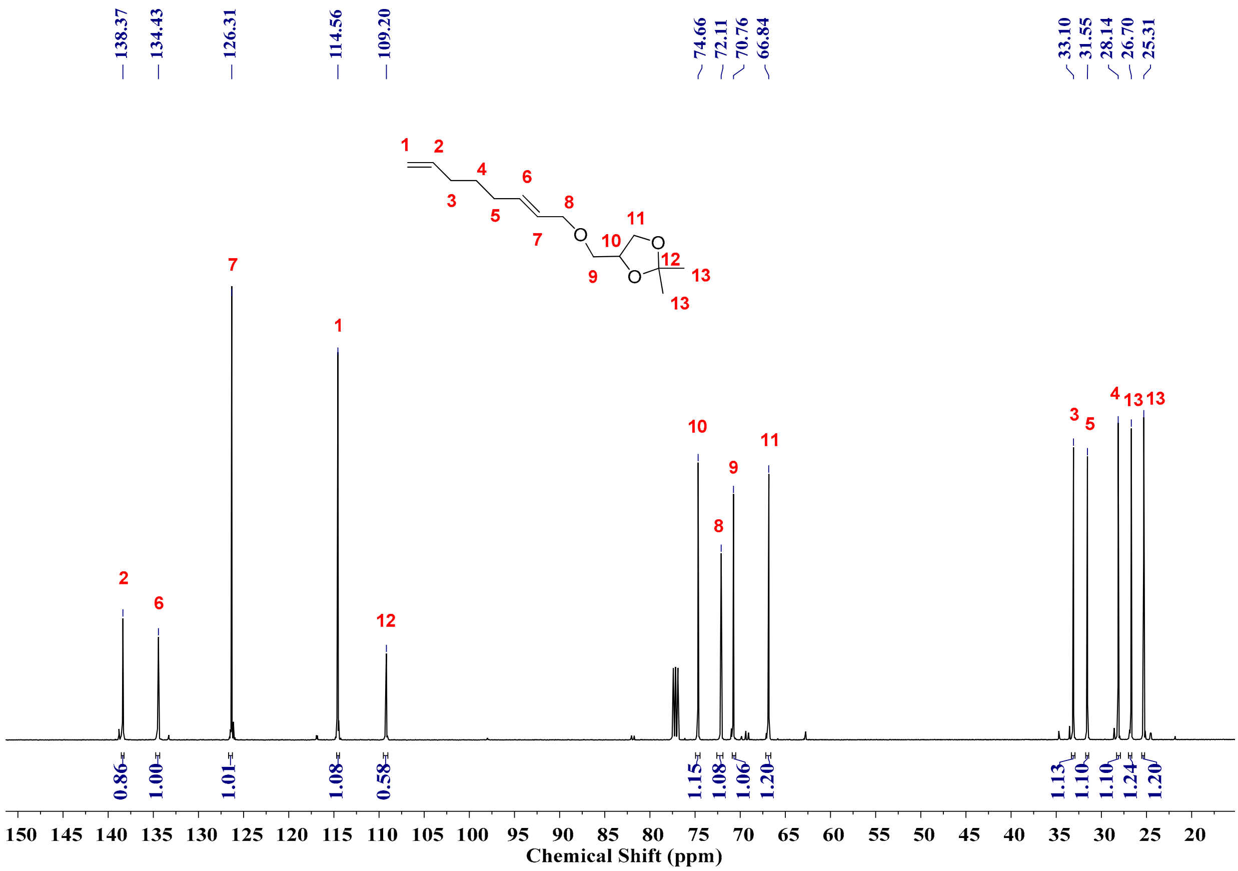

Figure S12. ^{13}C NMR spectrum of **OC8-SOL** in $CDCl_3$.

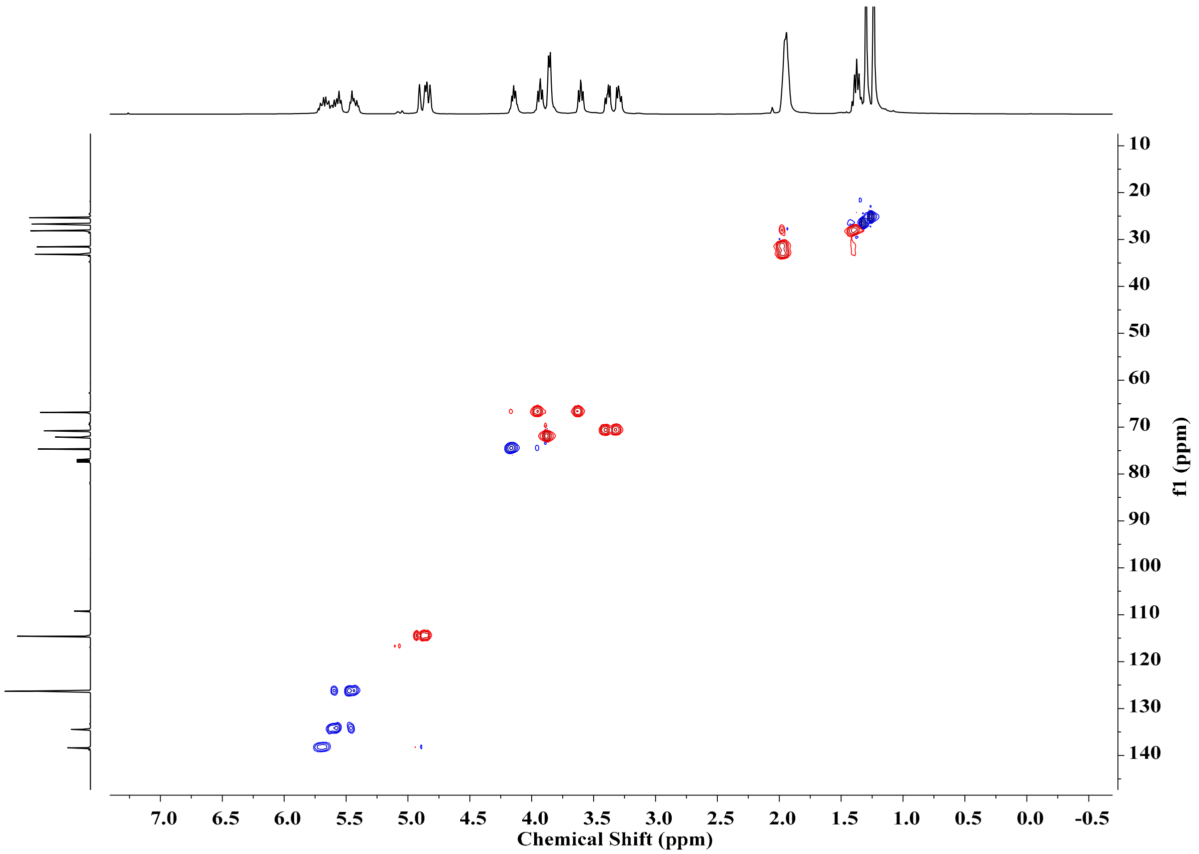

Figure S13. ^{1}H-^{13}C HSQC spectrum of **OC8-SOL** in $CDCl_3$.

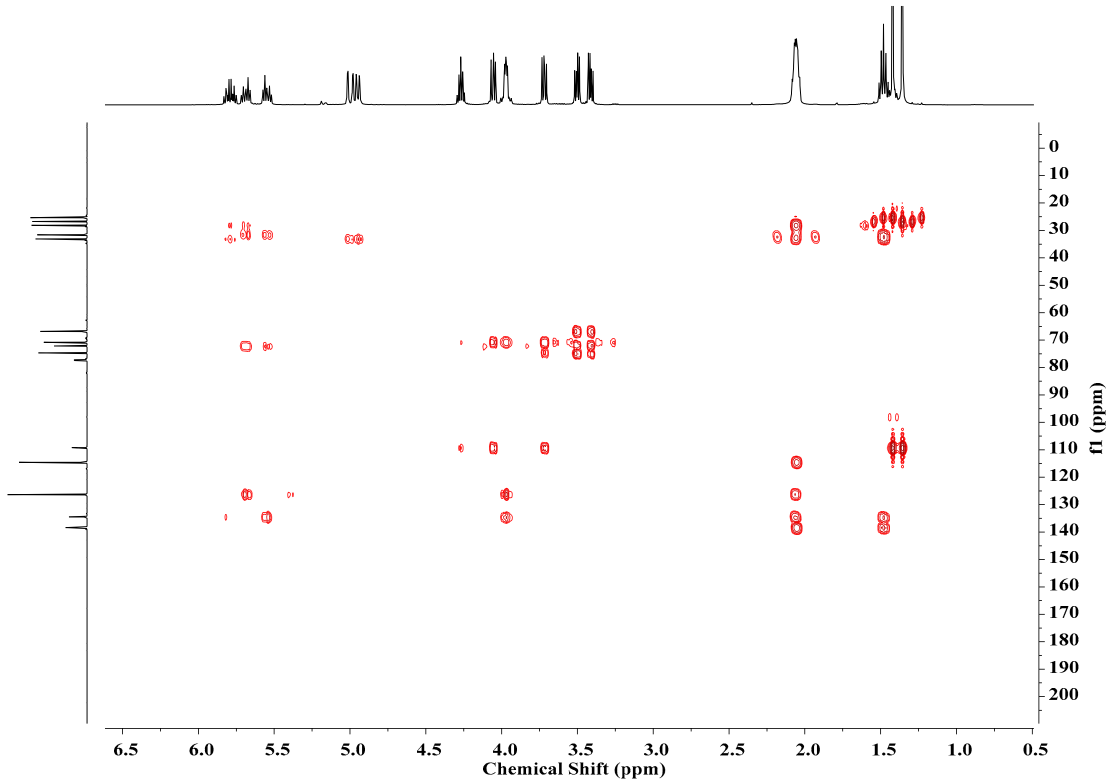

Figure S14. ^{1}H-^{13}C HMBC spectrum of **OC8-SOL** in $CDCl_3$.

3. NMR figures of copolymers

$$\text{brs} = \text{Me groups/1000C} = \frac{2*I_{Me}}{3*I_{tot}} * 1000 = \frac{2*3.60}{3*398.63} * 1000 = 6.0$$

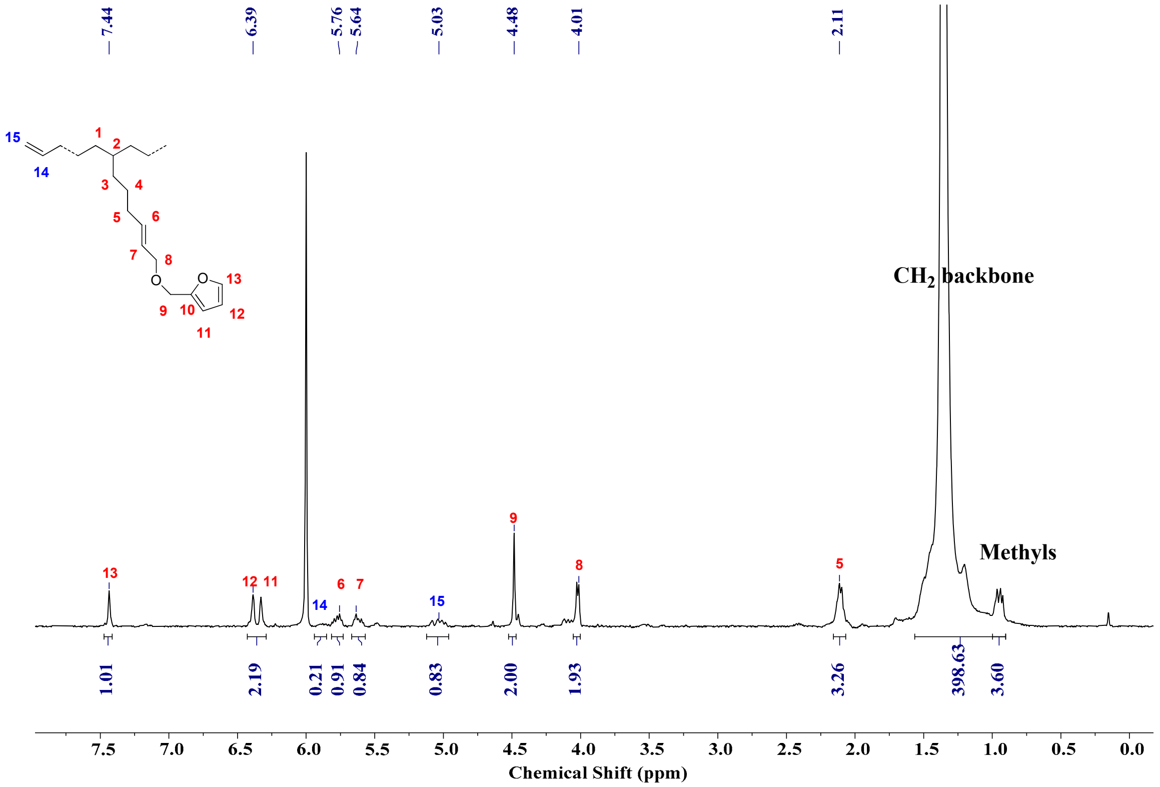

Figure S15. ^{1}H NMR spectrum (400 MHz, 110 °C, $C_2D_2Cl_4$) of copolymer from table 1, entry 3.

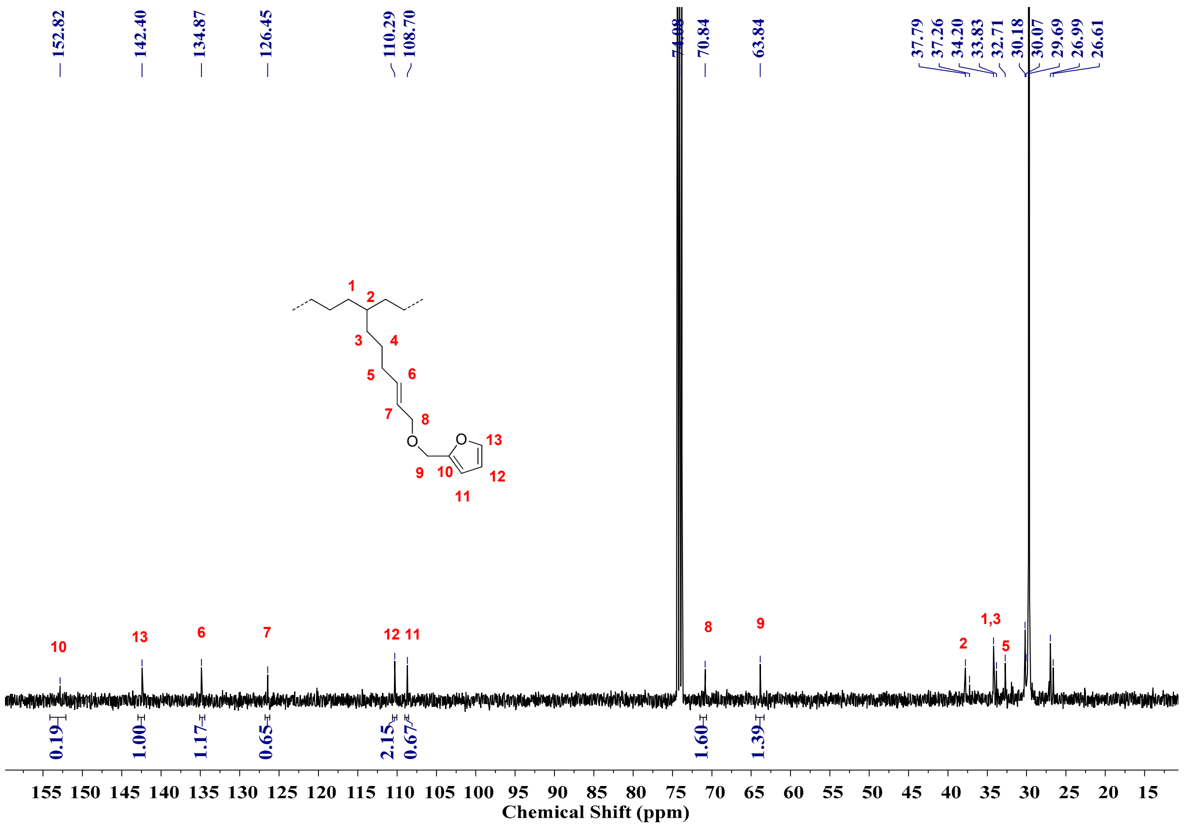

Figure S16. ^{13}C NMR spectrum (100 MHz, 110 °C, $C_2D_2Cl_4$) of copolymer from table 1, entry 3.

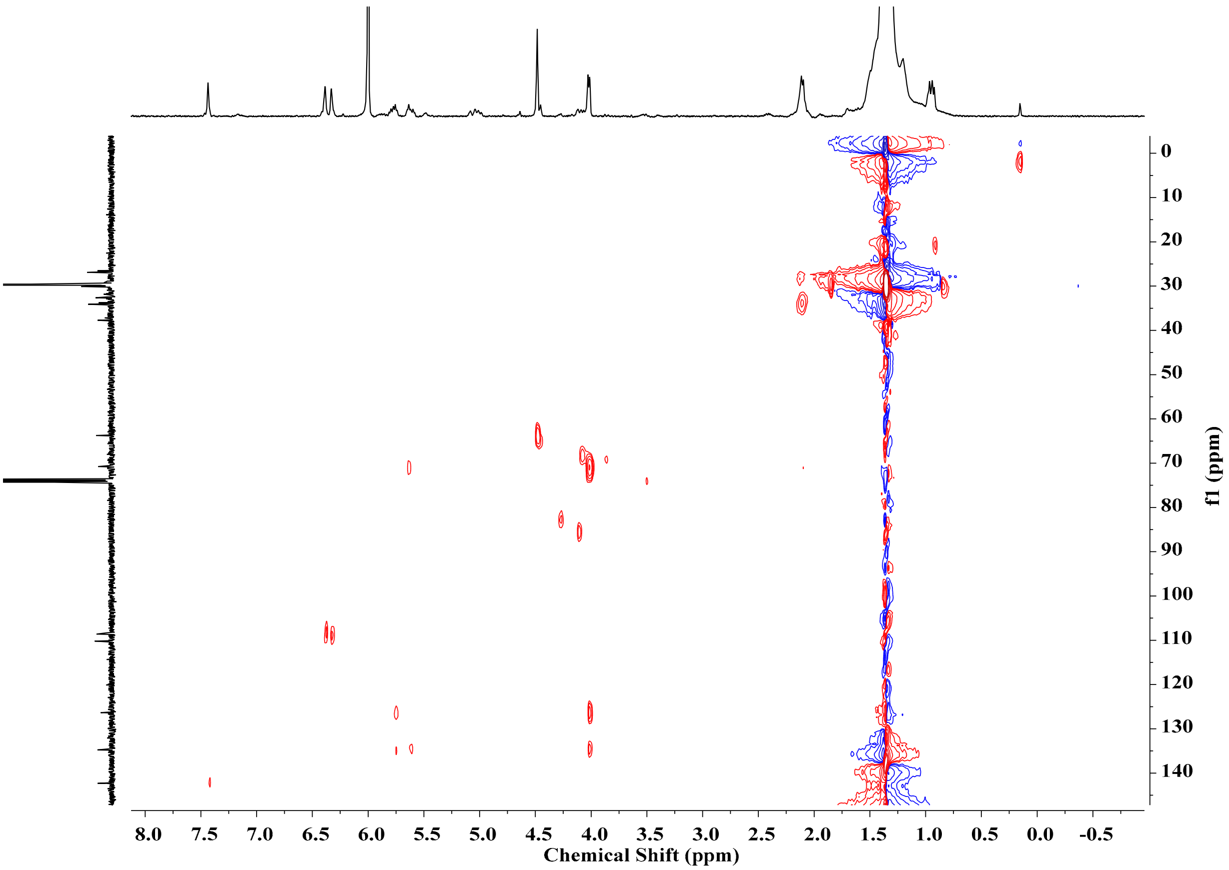

Figure S17. ^{1}H-^{13}C HSQC spectrum (110 °C, $C_2D_2Cl_4$) of copolymer from table 1, entry 3.

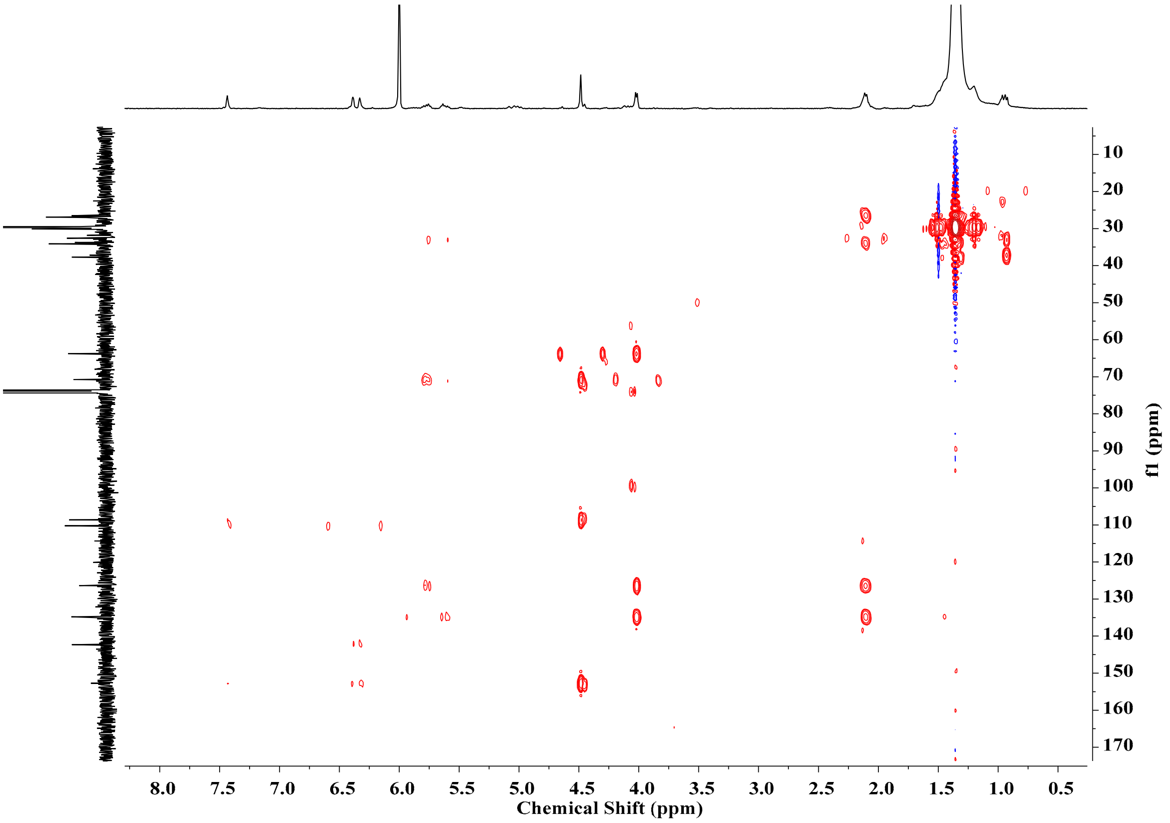

Figure S18. ^{1}H-^{13}C HMBC spectrum (110 °C, $C_2D_2Cl_4$) of copolymer from table 1, entry 3.

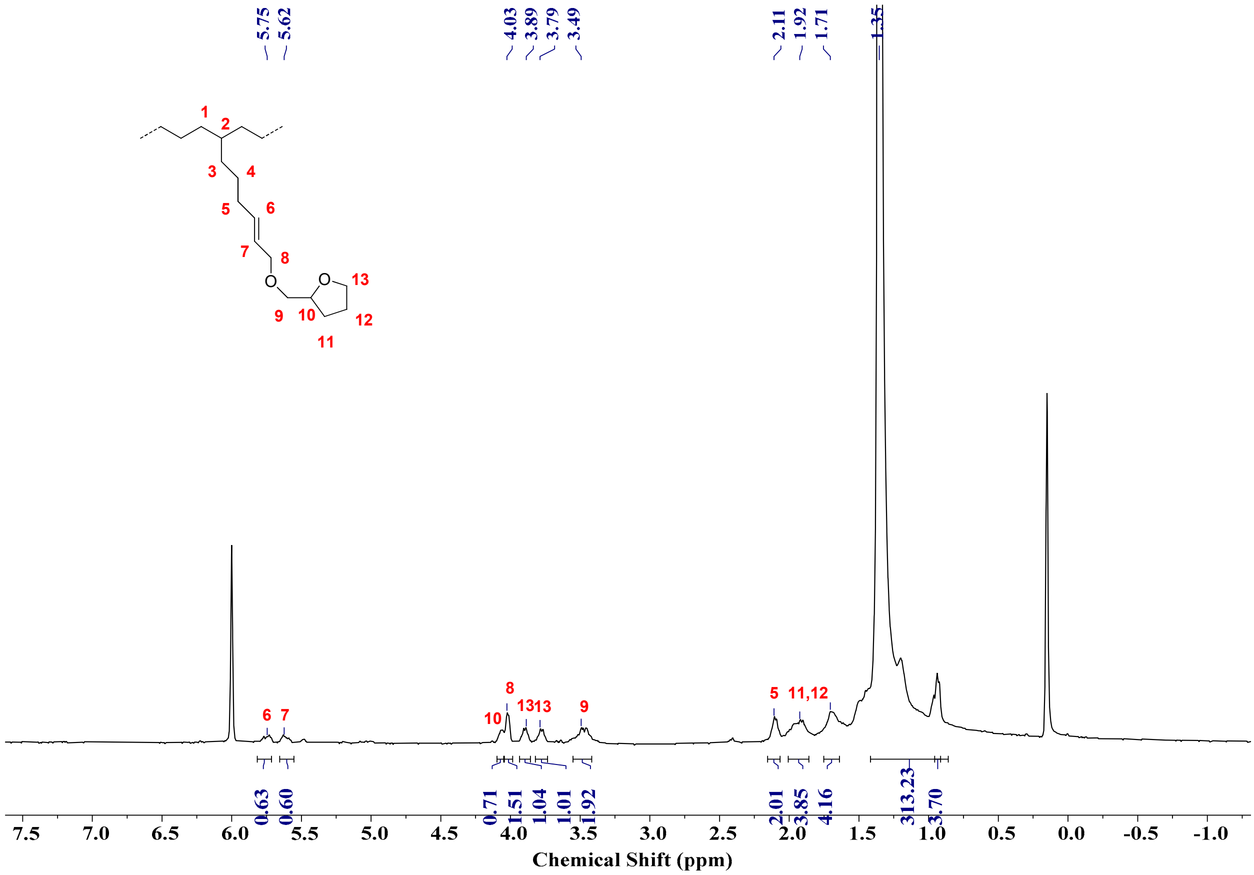

Figure S19. ^{1}H NMR spectrum (400 MHz, 110 °C, $C_2D_2Cl_4$) of copolymer from table 1, entry 4.

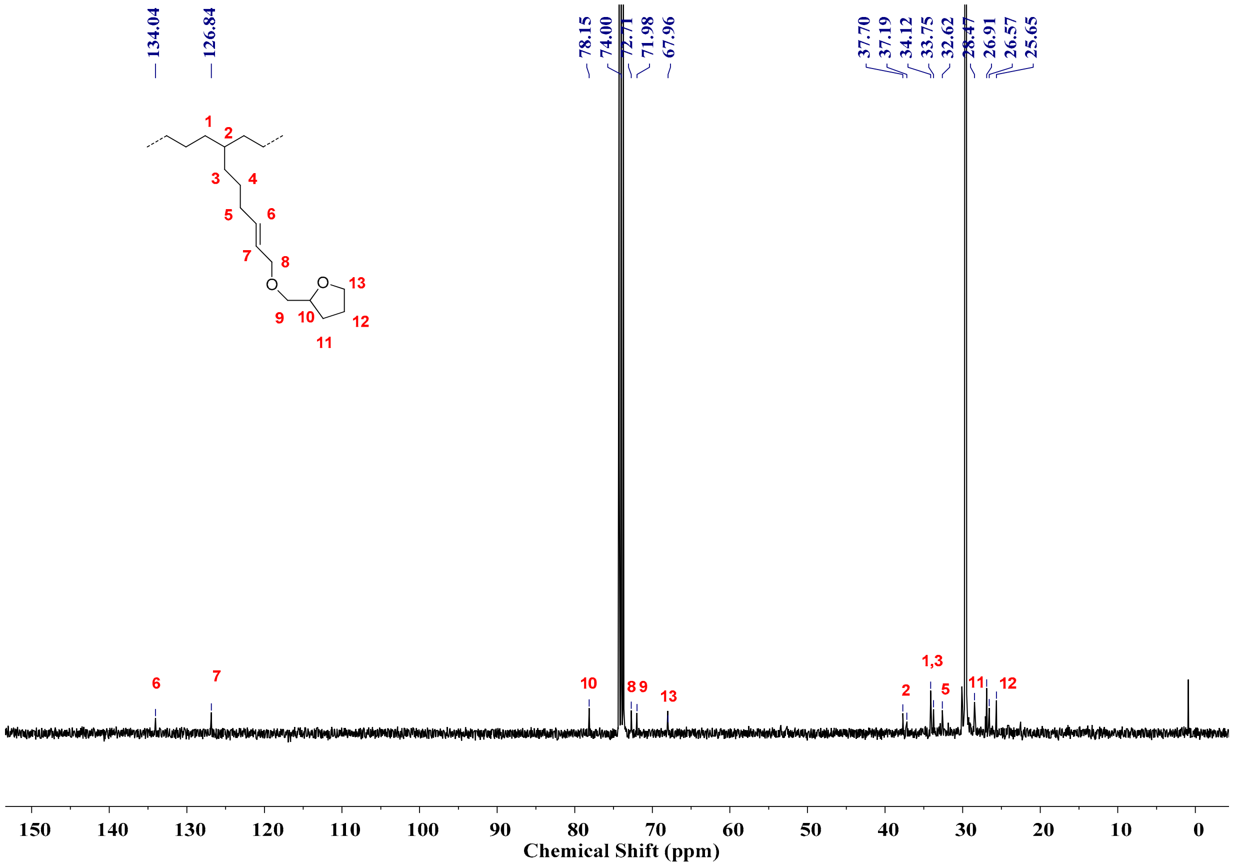

Figure S20. ^{13}C NMR spectrum (100 MHz, 110 °C, $C_2D_2Cl_4$) of copolymer from table 1, entry 4.

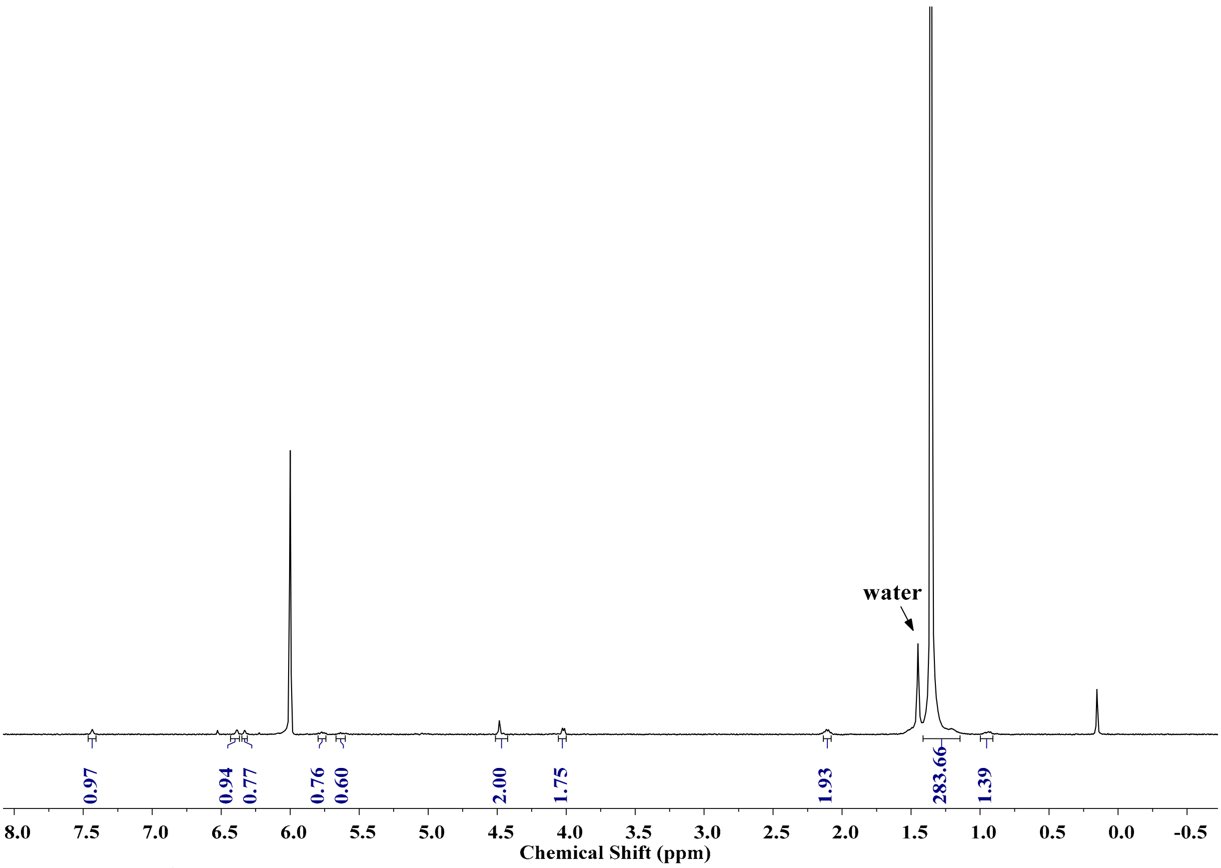

Figure S21. ^{1}H NMR spectrum (400 MHz, 110 °C, $C_2D_2Cl_4$) of copolymer from table 1, entry 7.

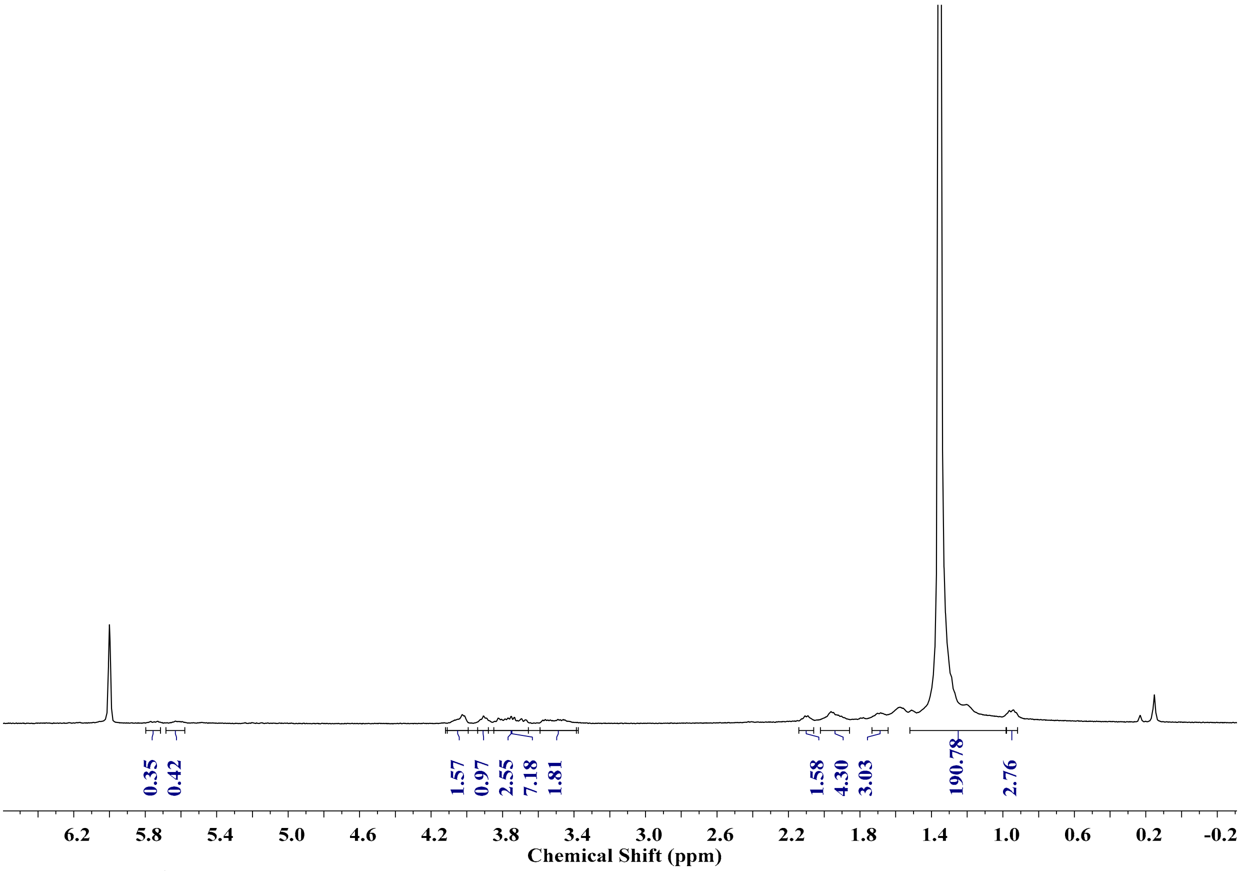

Figure S22 ^{1}H NMR spectrum (400 MHz, 110 °C, $C_2D_2Cl_4$) of copolymer from table 1, entry 9.

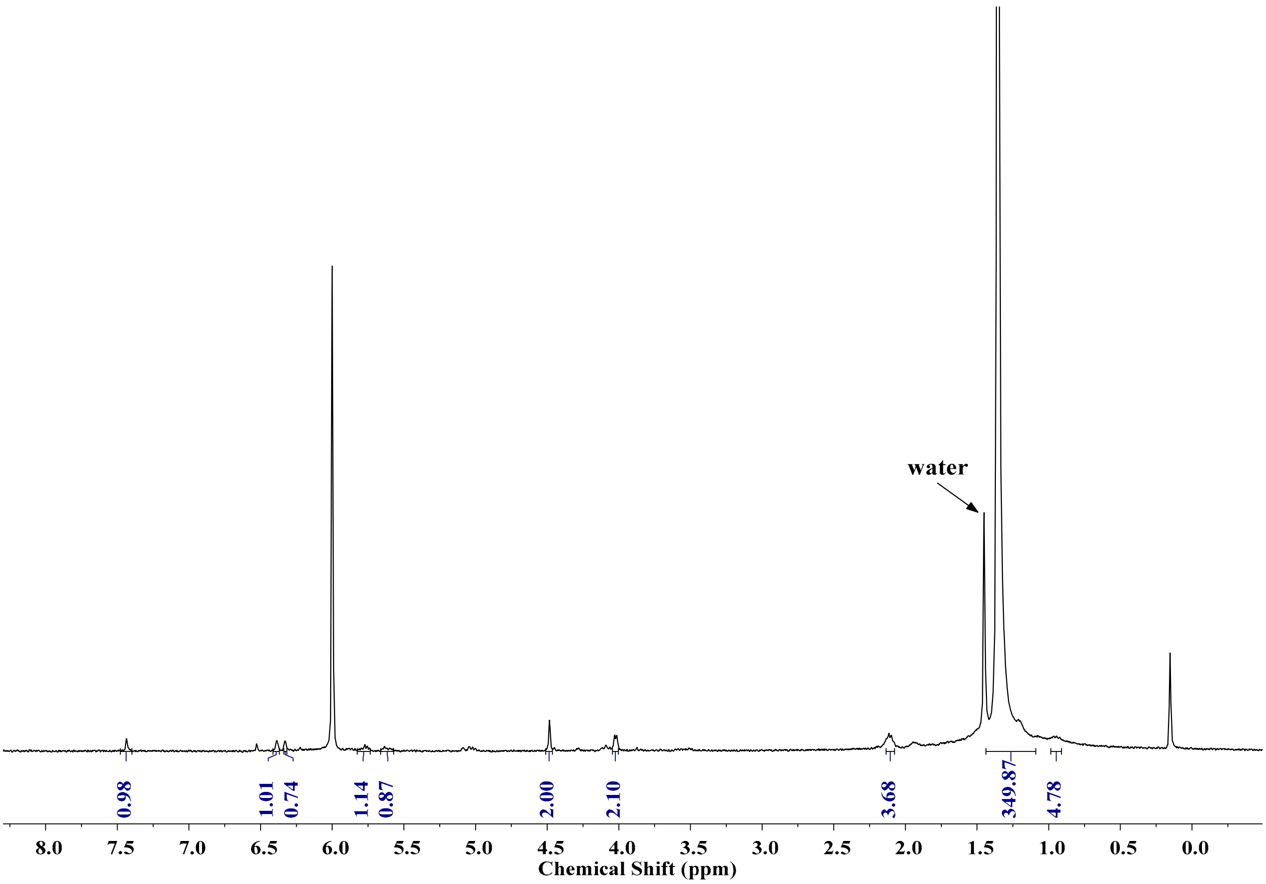

Figure S23. ^{1}H NMR spectrum (400 MHz, 110 °C, $C_2D_2Cl_4$) copolymer from table 1, entry 13.

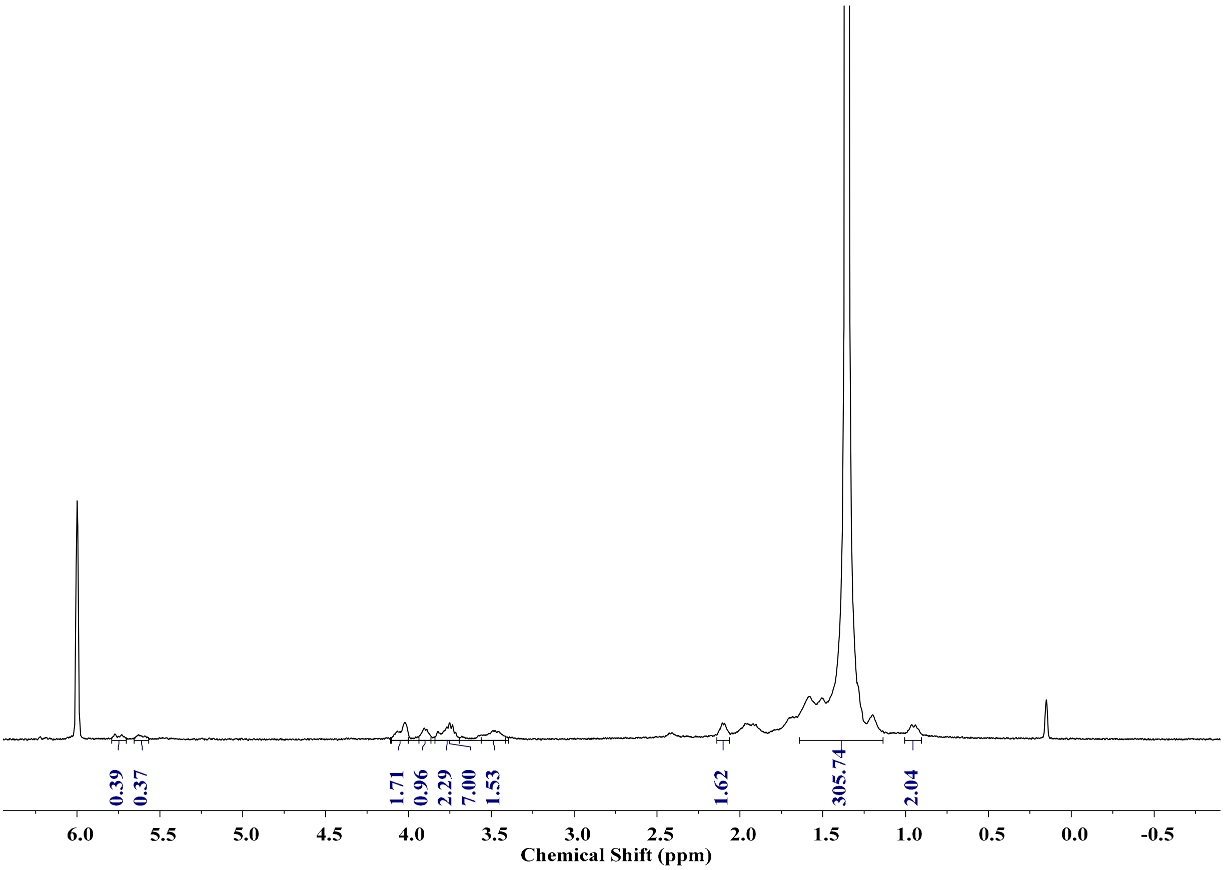

Figure S24. ^{1}H NMR spectrum (400 MHz, 110 °C, $C_2D_2Cl_4$) copolymer from table 1, entry 14.

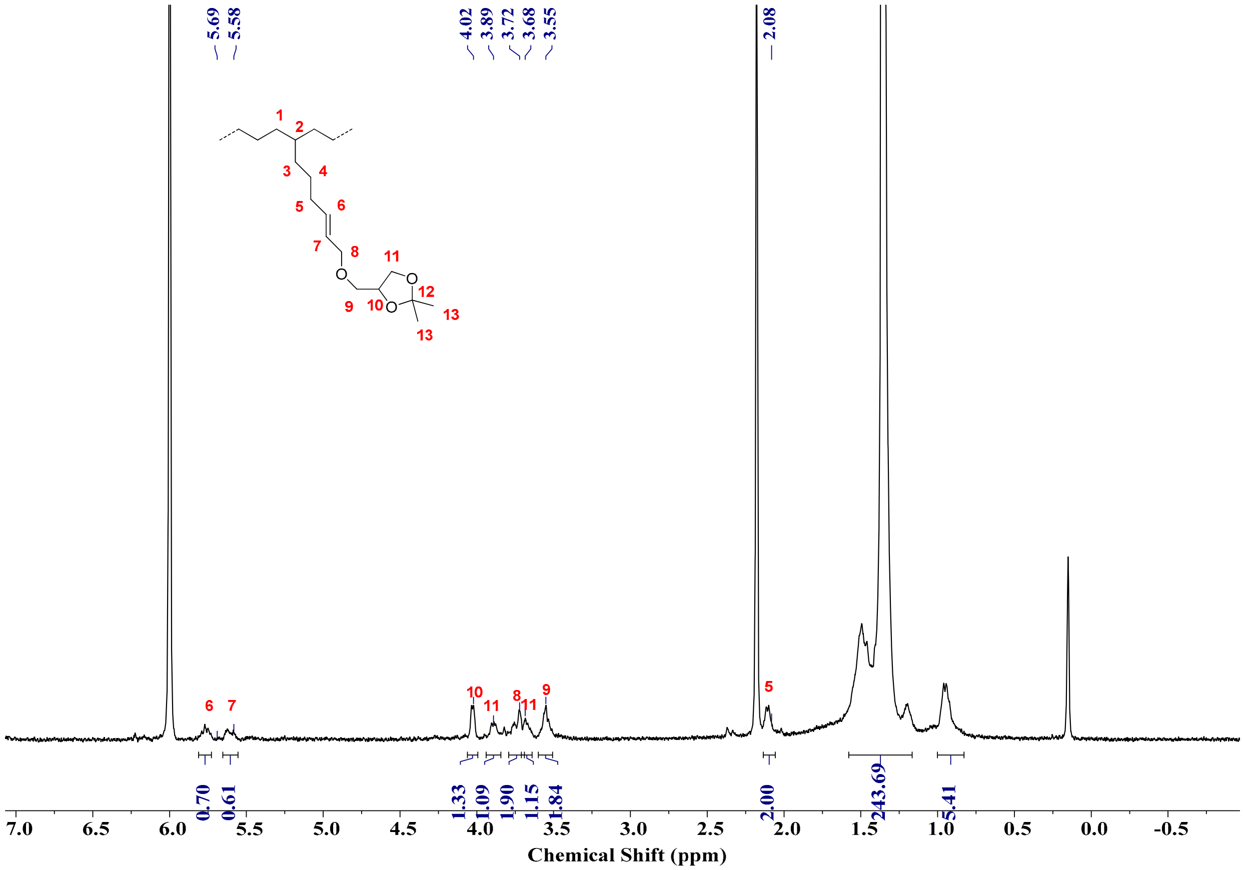

Figure S25. ^{1}H NMR spectrum (400 MHz, 110 °C, $C_2D_2Cl_4$) of copolymer from table 1, entry 15.

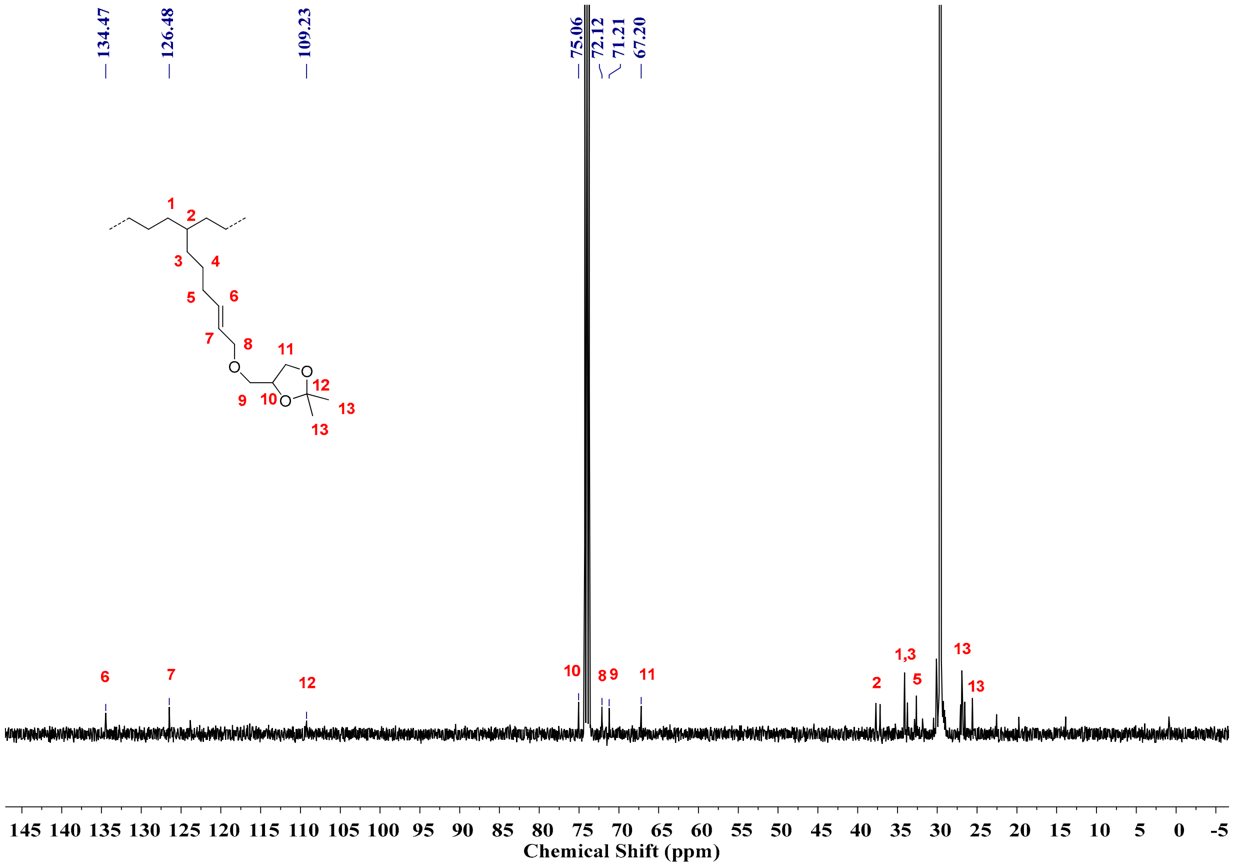

Figure S26. ^{13}C NMR spectrum (100 MHz, 110 °C, $C_2D_2Cl_4$) of copolymer from table 1, entry 15.

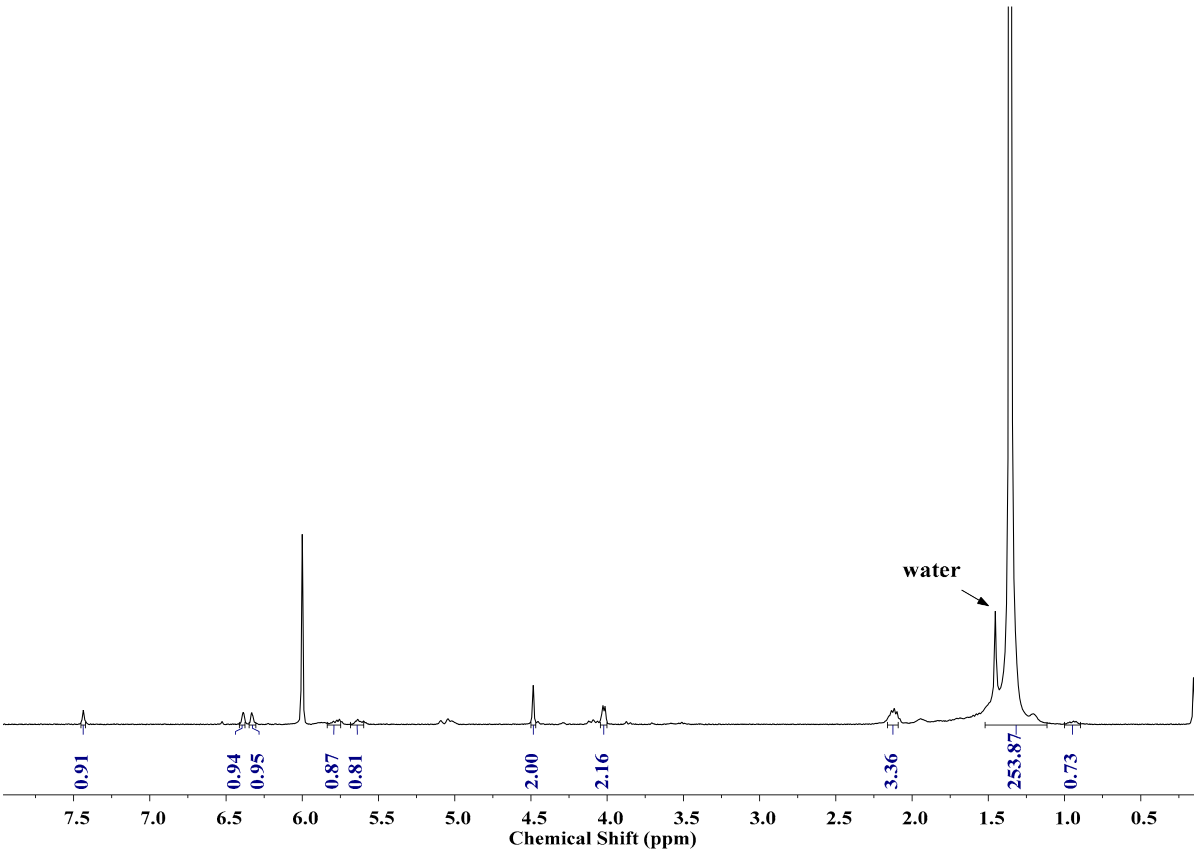

Figure S27. ^{1}H NMR spectrum (400 MHz, 110 °C, $C_2D_2Cl_4$) copolymer from table 1, entry 17.

4. GPC figures of copolymers

MW Averages

Mp: 19494	Mn: 11844	Mv: 20430	Mw: 22032
Mz: 35950	Mz+1: 51599	PD: 1.8602	

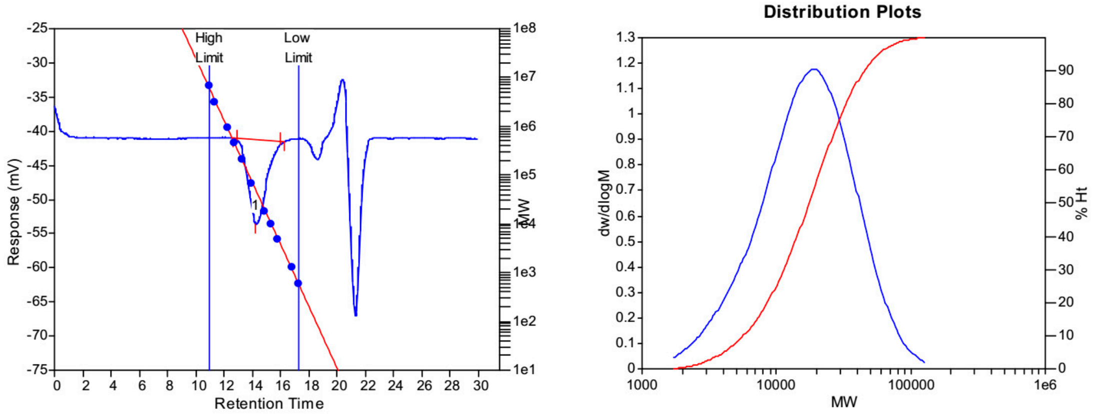

Figure S28. GPC trace of the polymer from table 1, entry 1.

Mp: 12851	Mn: 7235	Mv: 14508	Mw: 15965
Mz: 30487	Mz+1: 52769	PD: 2.2066	

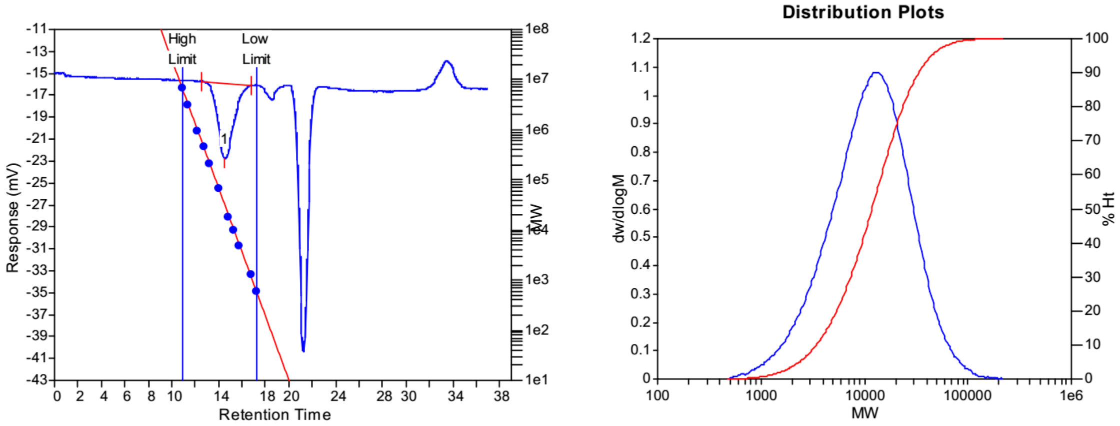

Figure S29. GPC trace of the polymer from table 1, entry 3.

Mp: 12254	Mn: 6715	Mv: 13256	Mw: 14516
Mz: 26763	Mz+1: 45471	PD: 2.1617	

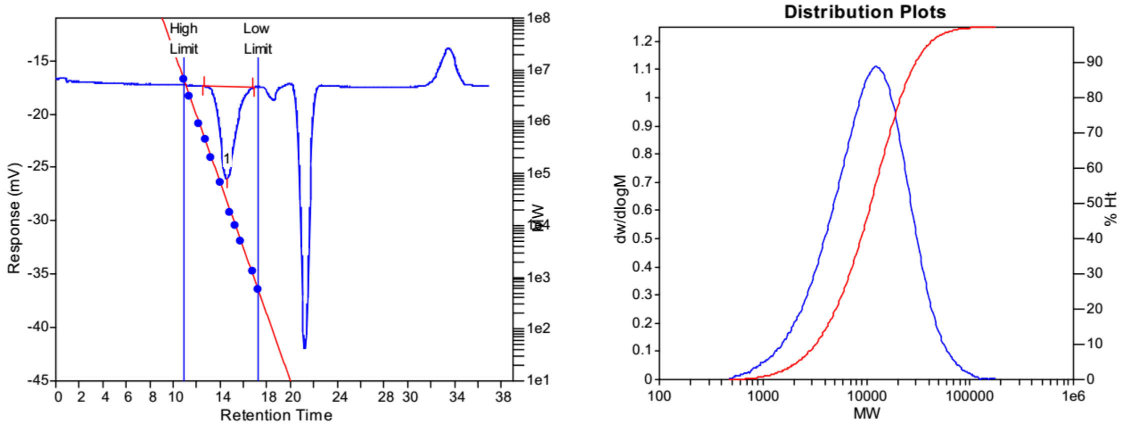

Figure S30. GPC trace of the polymer from table 1, entry 4.

MW Averages

Mp: 12549	Mn: 6515	Mv: 12873	Mw: 14079
Mz: 25412	Mz+1: 40926	PD: 2.1610	

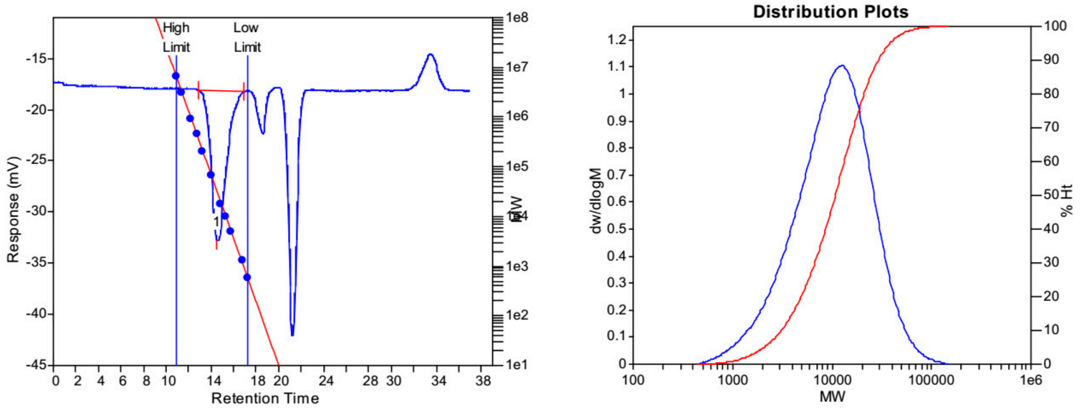

Figure S31. GPC trace of the polymer from table 1, entry 5.

MW Averages

Mp: 37815	Mn: 22456	Mv: 43593	Mw: 47938
Mz: 89986	Mz+1: 144388	PD: 2.1348	

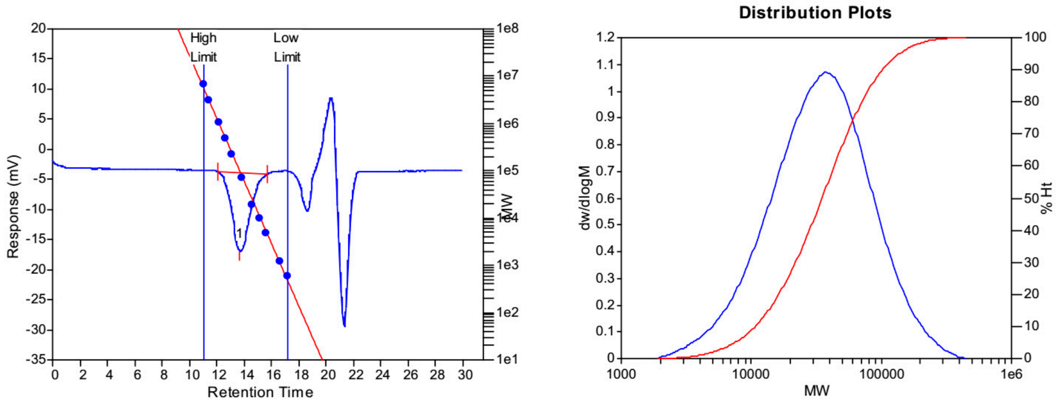

Figure S32. GPC trace of the polymer from table 1, entry 8.

MW Averages

Mp: 26790	Mn: 15783	Mv: 30458	Mw: 33396
Mz: 62781	Mz+1: 109686	PD: 2.1159	

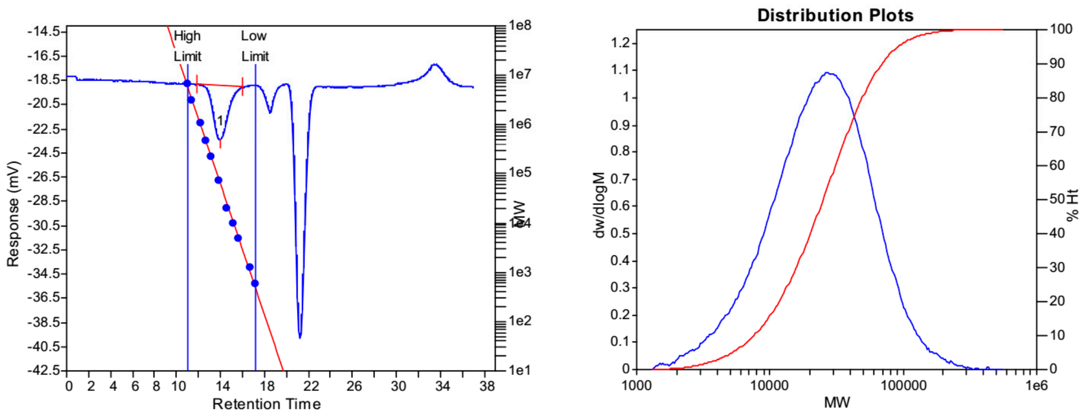

Figure S33. GPC trace of the polymer from table 1, entry 9.

MW Averages

Mp: 53479 Mn: 28184 Mv: 55946 Mw: 61007

Mz: 107582 Mz+1: 168627 PD: 2.1646

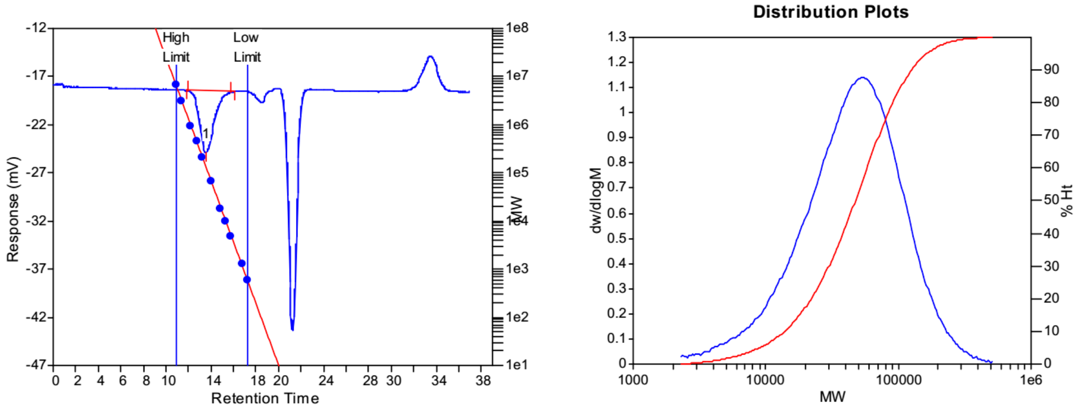

Figure S34. GPC trace of the polymer from table 1, entry 10.

MW Averages

Mp: 66203 Mn: 40233 Mv: 69988 Mw: 75242

Mz: 118483 Mz+1: 161035 PD: 1.8702

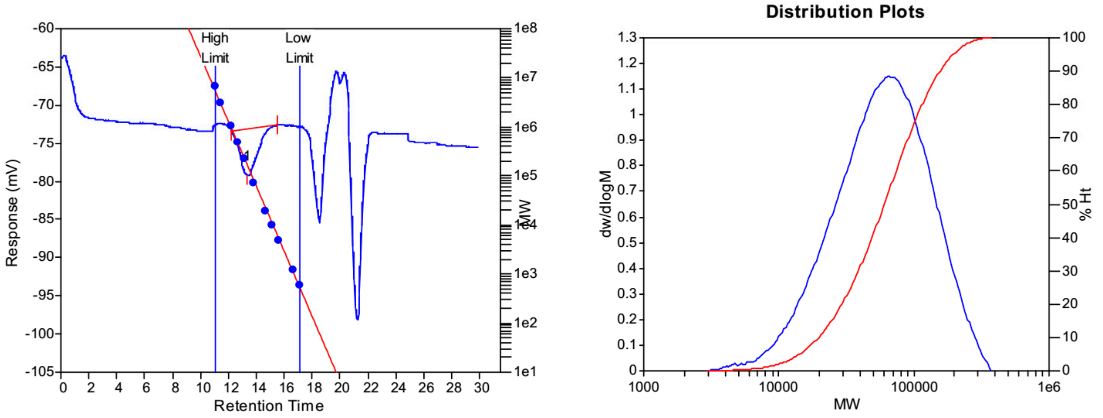

Figure S35. GPC trace of the polymer from table 1, entry 12.

MW Averages

Mp: 50813 Mn: 31121 Mv: 55645 Mw: 60510

Mz: 105481 Mz+1: 161228 PD: 1.9443

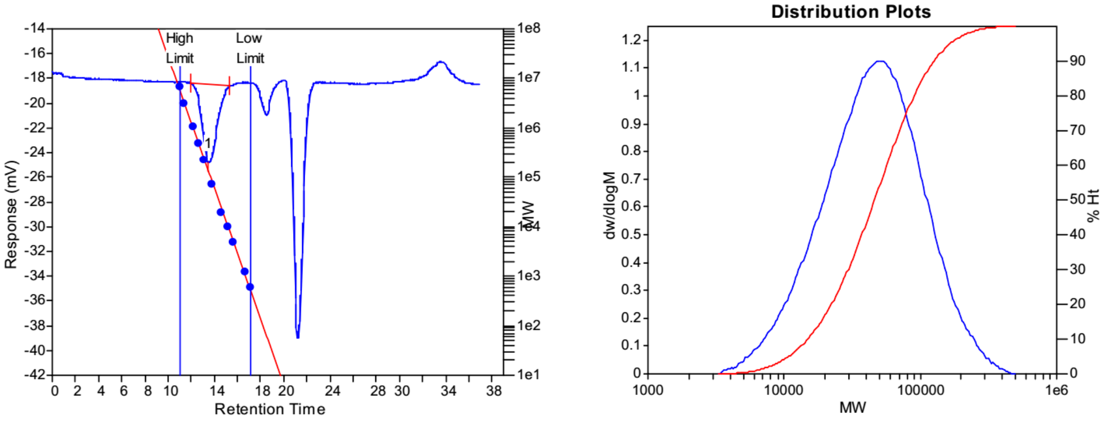

Figure S36. GPC trace of the polymer from table 1, entry 14.

MW Averages

Mp: 50813	Mn: 27821	Mv: 54508	Mw: 59771
Mz: 110387	Mz+1: 180459	PD: 2.1484	

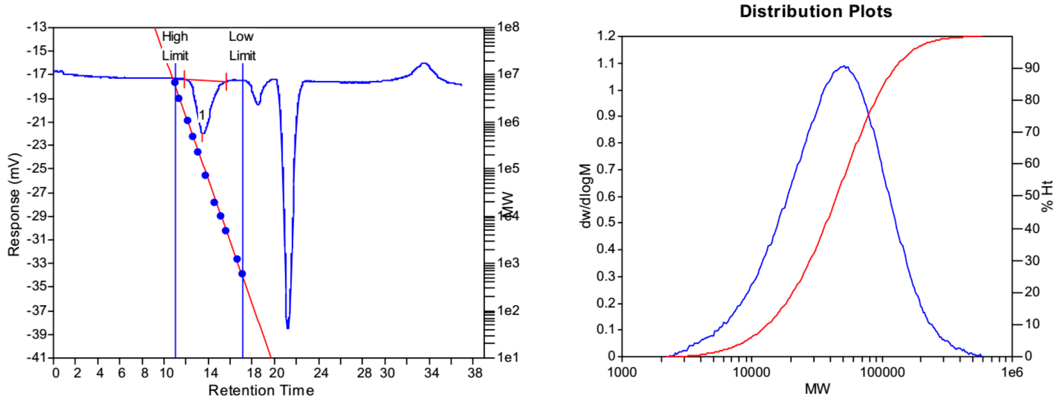

Figure S37. GPC trace of the polymer from table 1, entry 15.

MW Averages

Mp: 76612	Mn: 56023	Mv: 87900	Mw: 94208
Mz: 151068	Mz+1: 221259	PD: 1.6816	

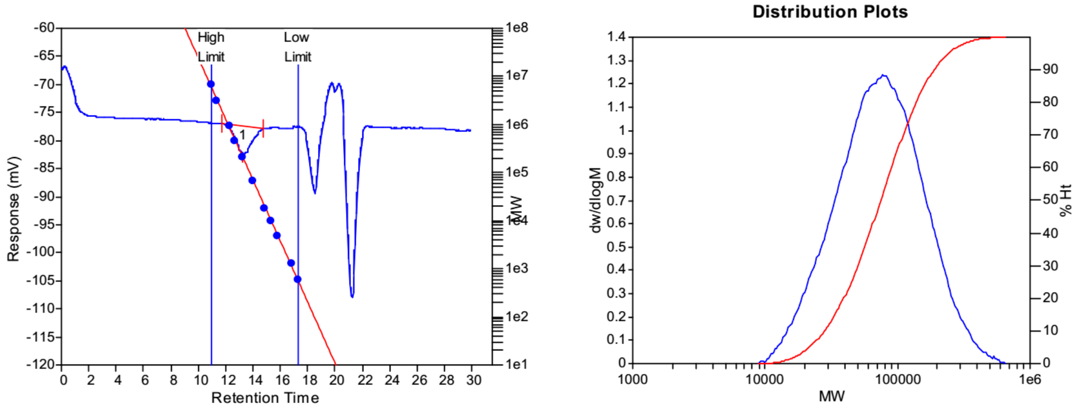

Figure S38. GPC trace of the polymer from table 1, entry 16.

MW Averages

Mp: 22549	Mn: 14063	Mv: 25370	Mw: 27508
Mz: 45396	Mz+1: 62619	PD: 1.9561	

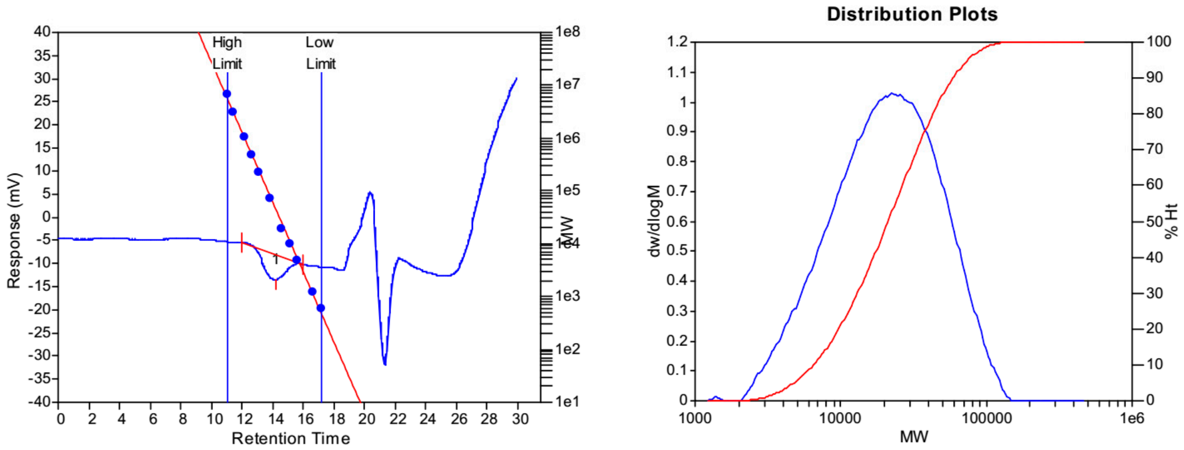

Figure S39. GPC trace of the polymer from table 1, entry 17.

5. DSC figures of copolymers

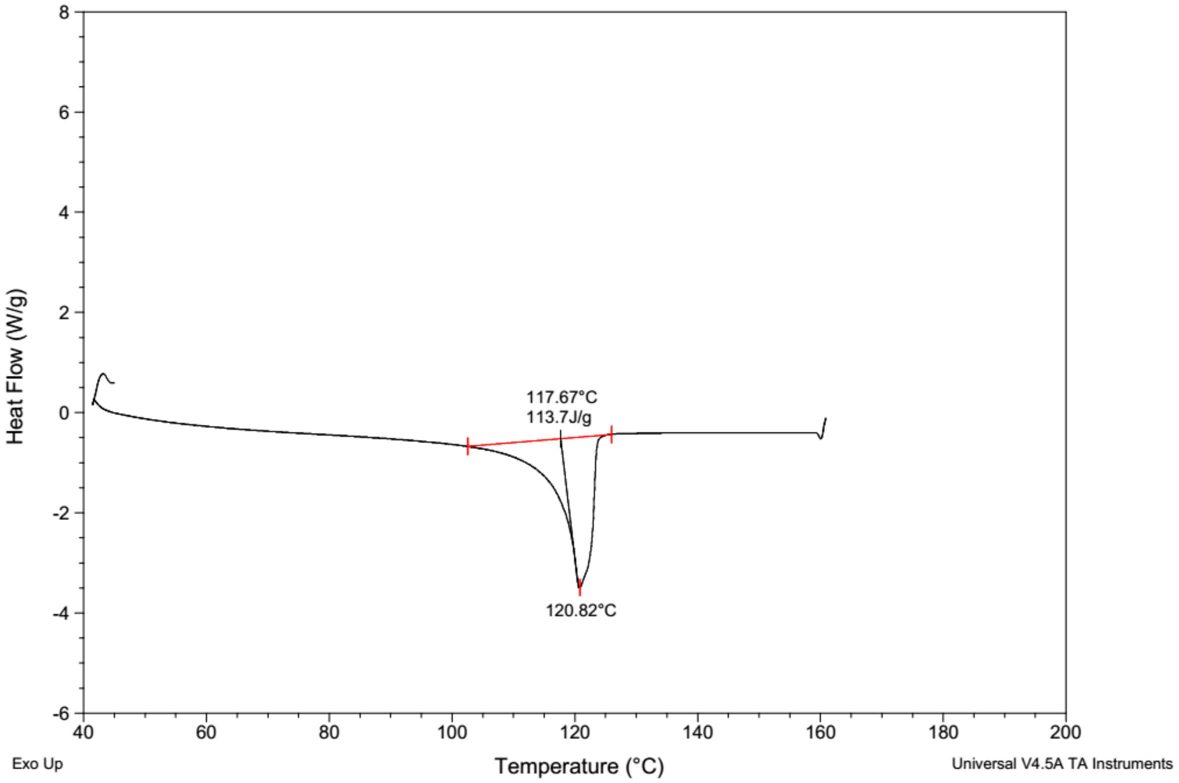

Figure S40. DSC data of the polymer from table 1, entry 2.

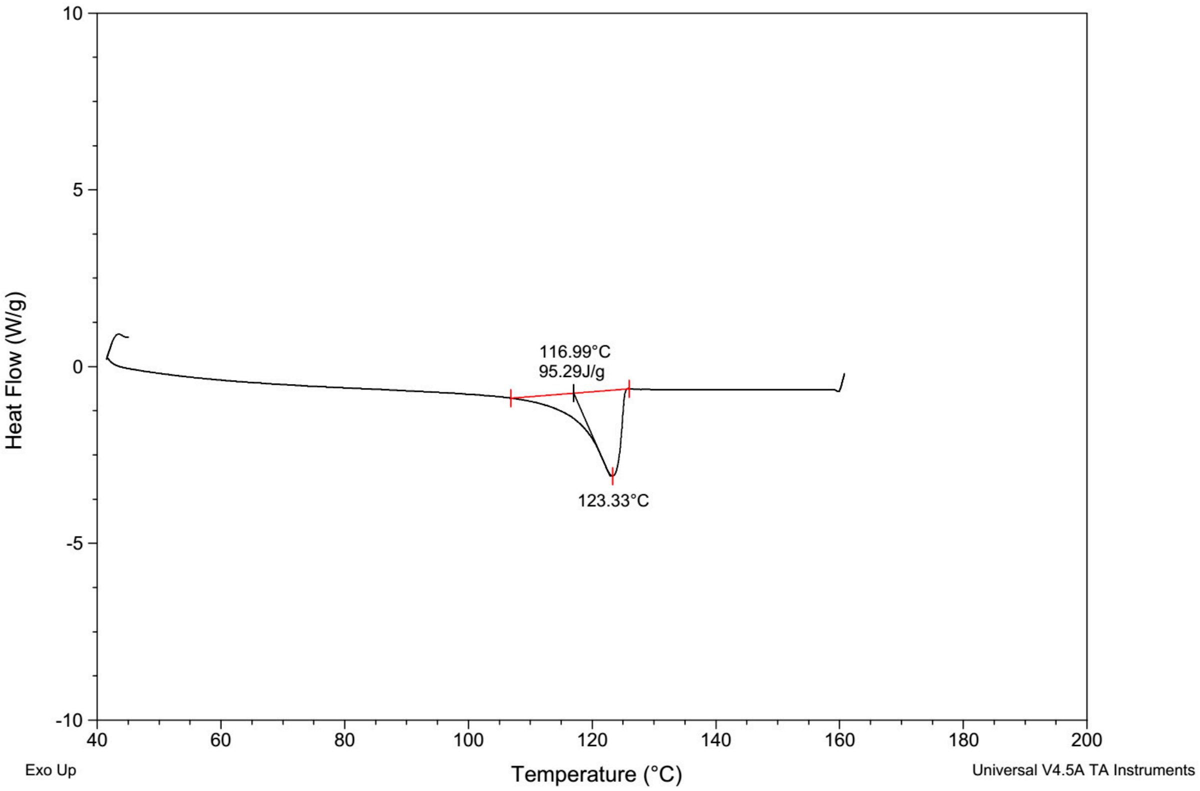

Figure S41. DSC data of the polymer from table 1, entry 6.

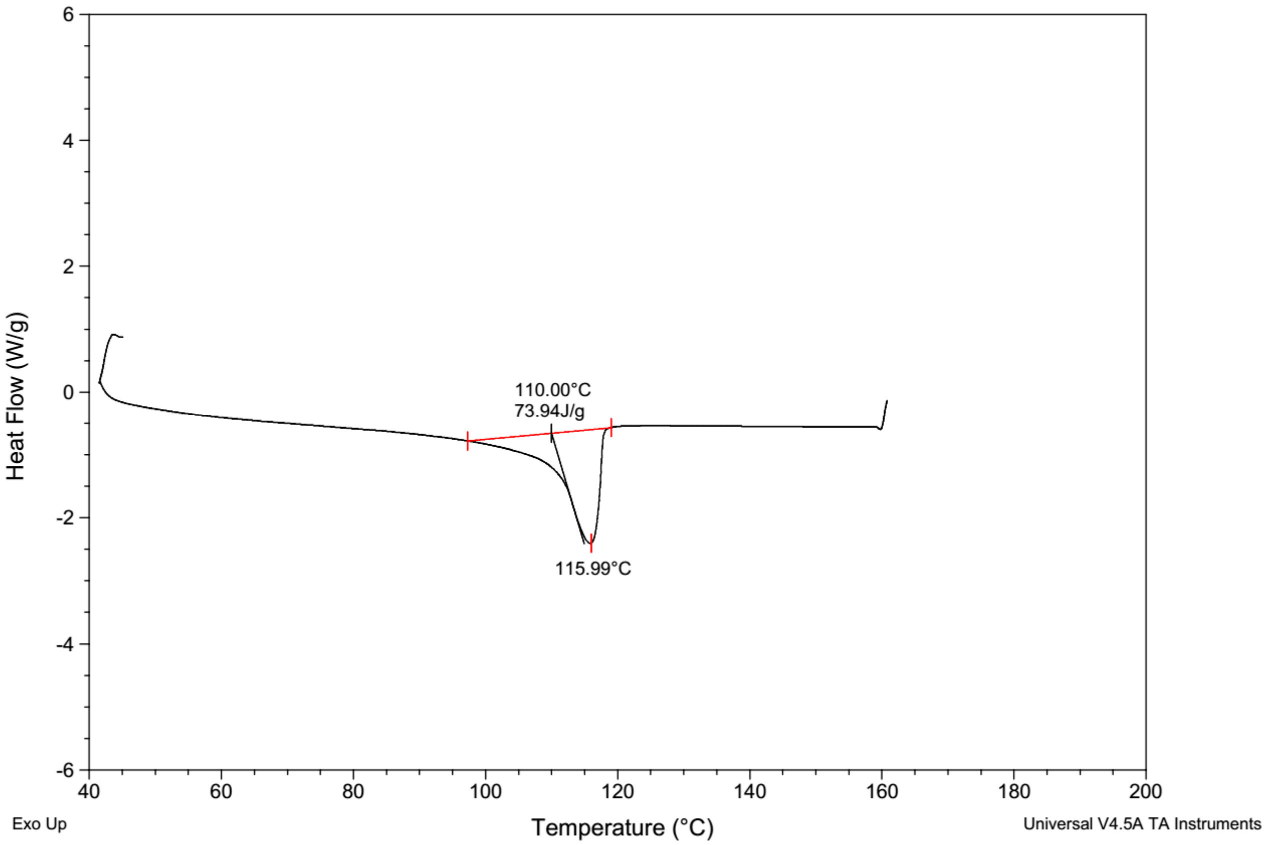

Figure S42. DSC data of the polymer from table 1, entry 7.

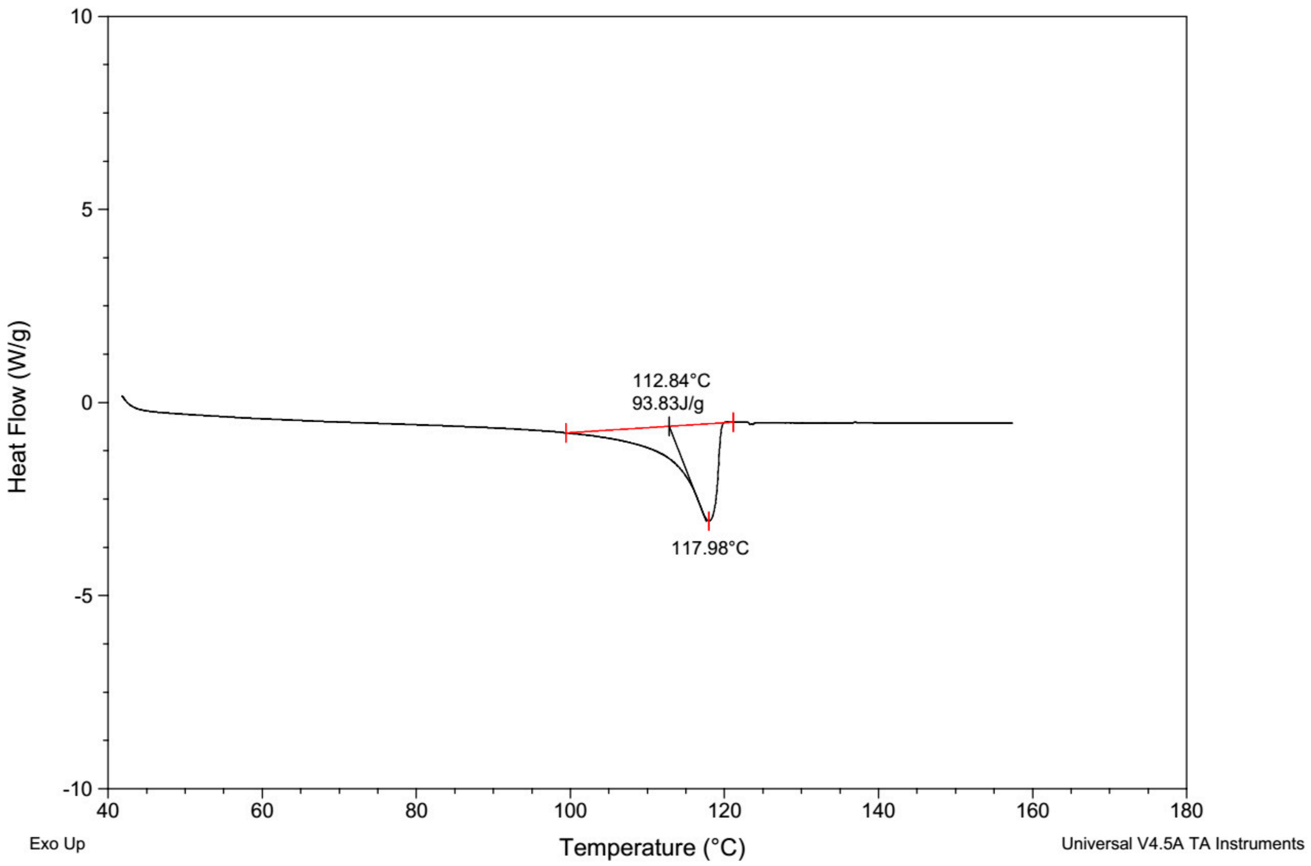

Figure S43. DSC data of the polymer from table 1, entry 12.

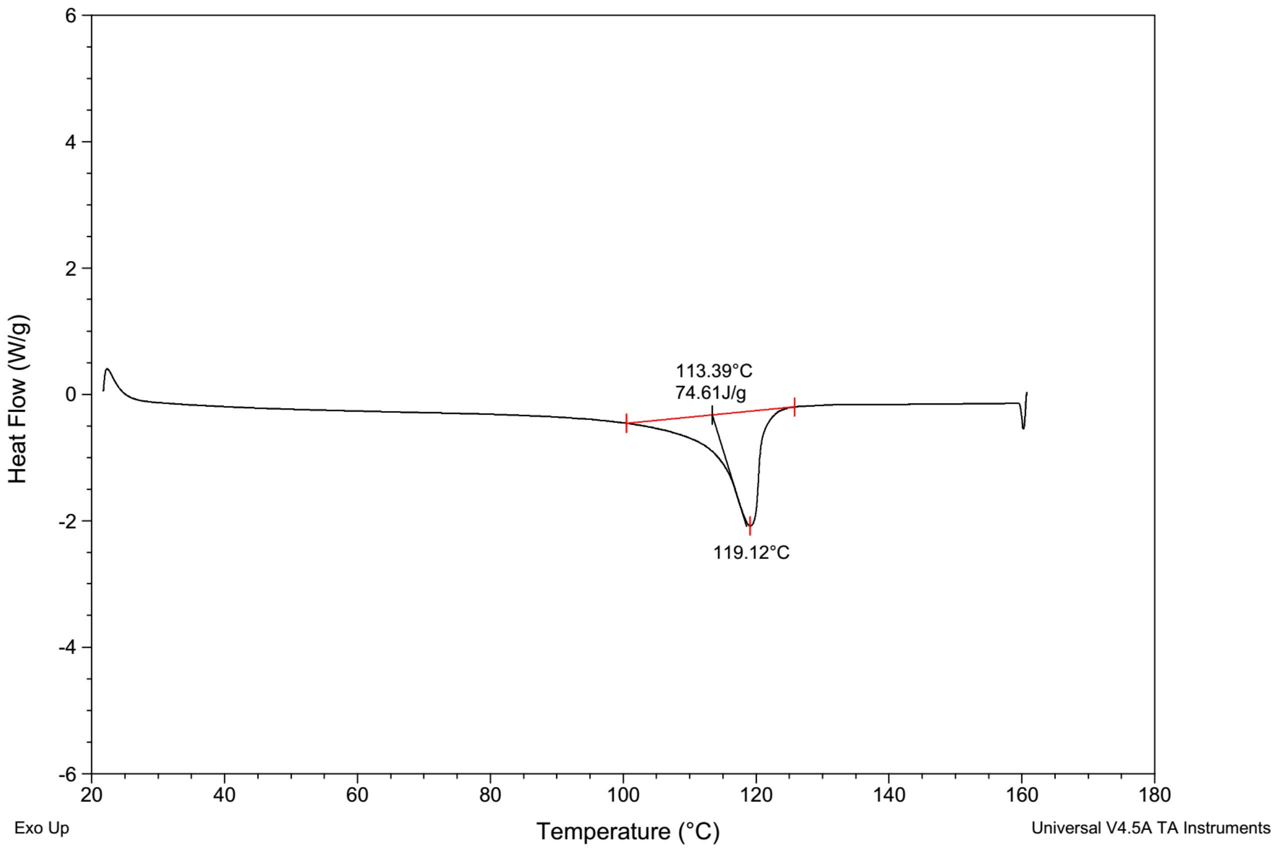

Figure S44. DSC data of the polymer from table 1, entry 14.

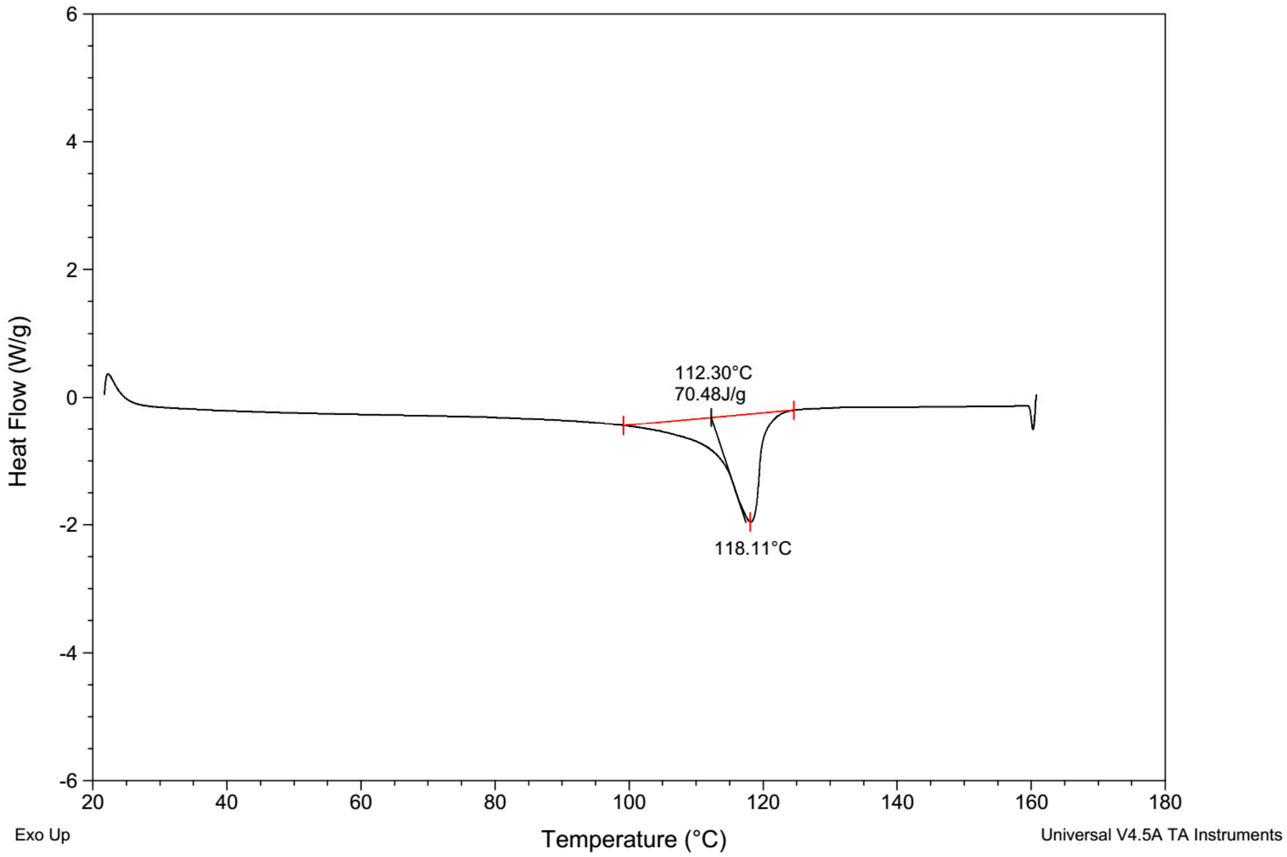

Figure S45. DSC data of the polymer from table 1, entry 15.

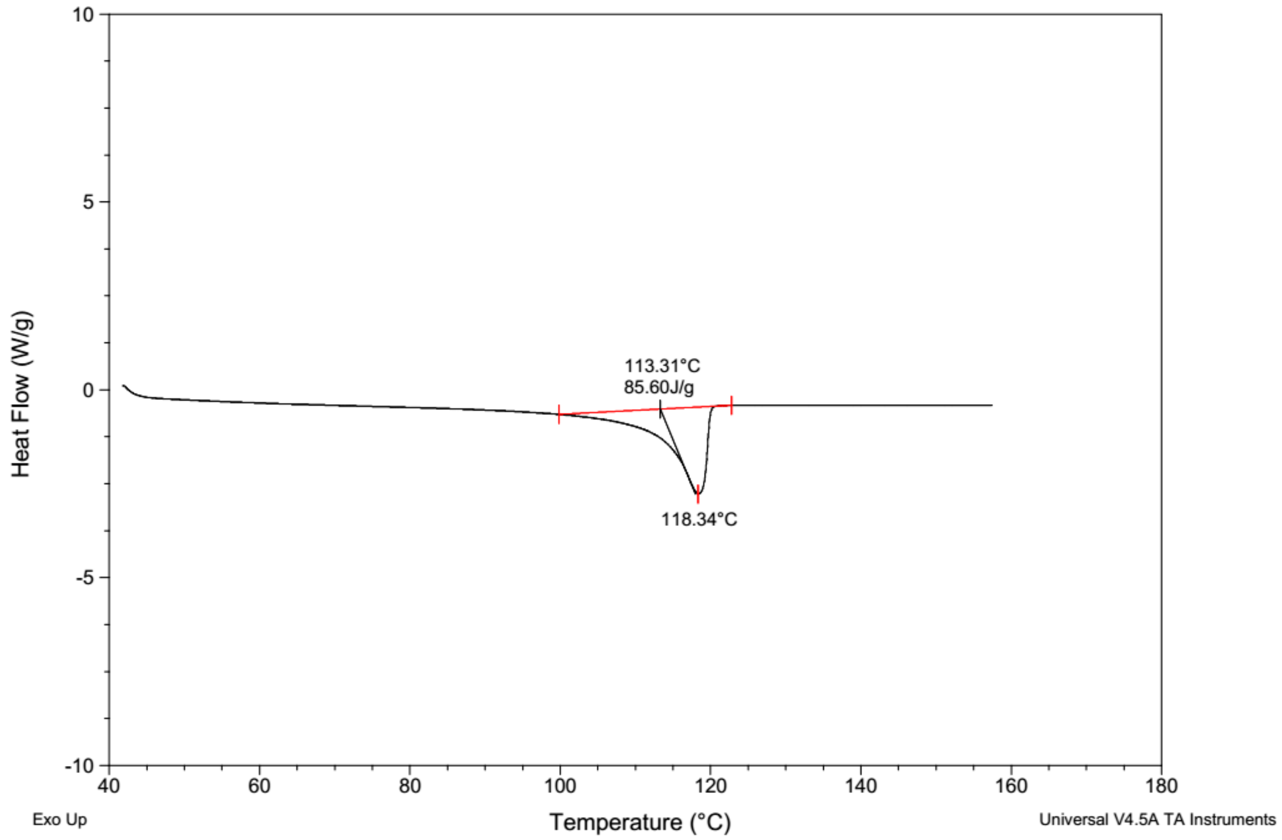

Figure S46. DSC data of the polymer from table 1, entry 16.

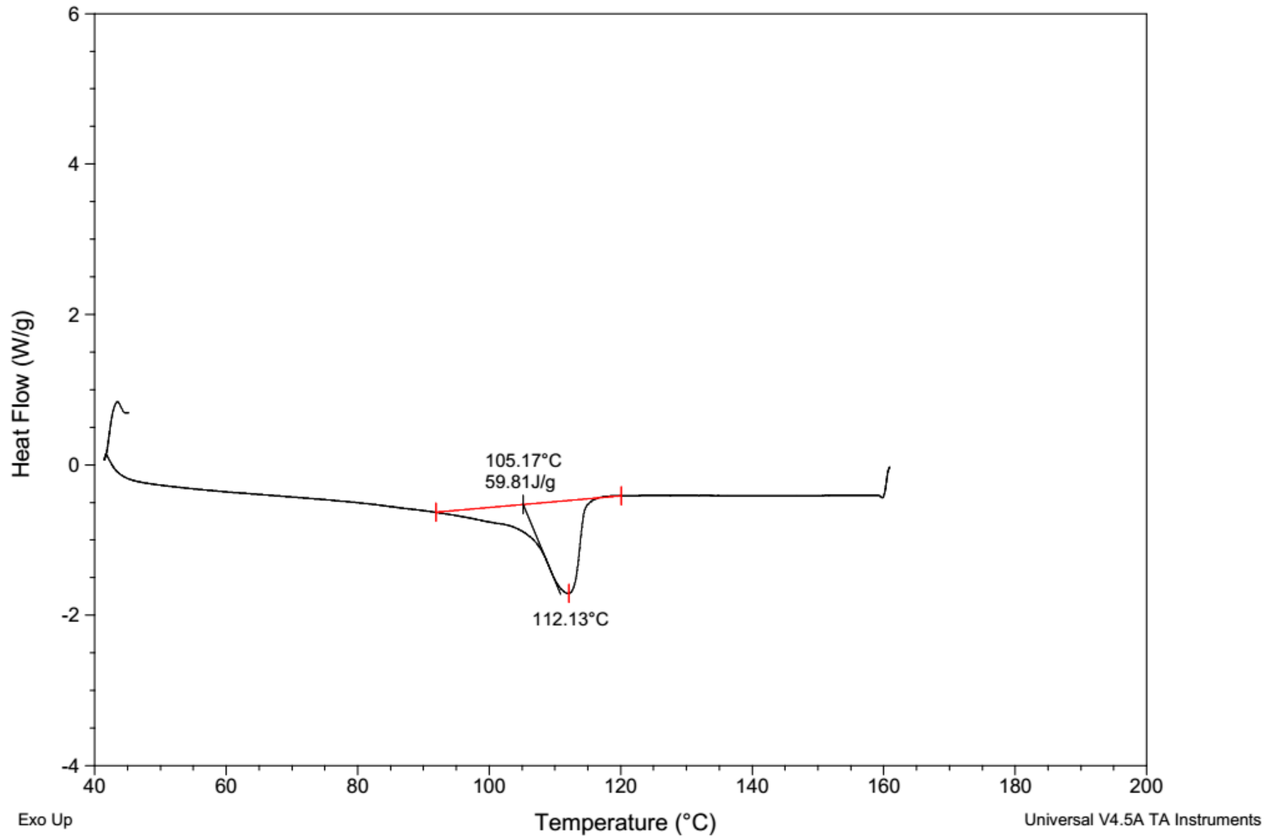

Figure S47. DSC data of the polymer from table 1, entry 17.

6. Steric maps of Pd-1, Pd-2 and Pd-3

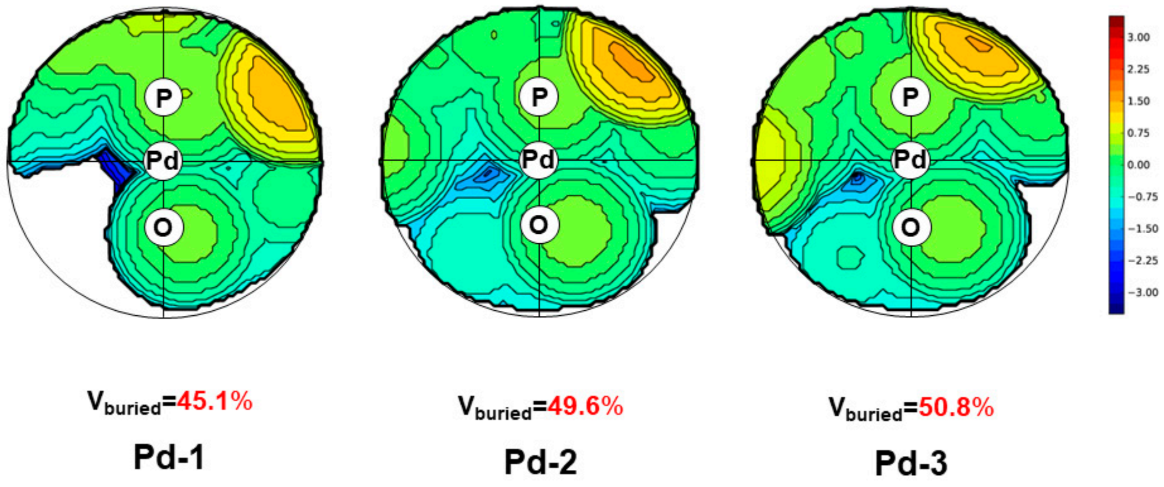

Figure S48. Steric maps of **Pd-1**, **Pd-2** and **Pd-3**.

7. Possible reaction pathways in the copolymerization process

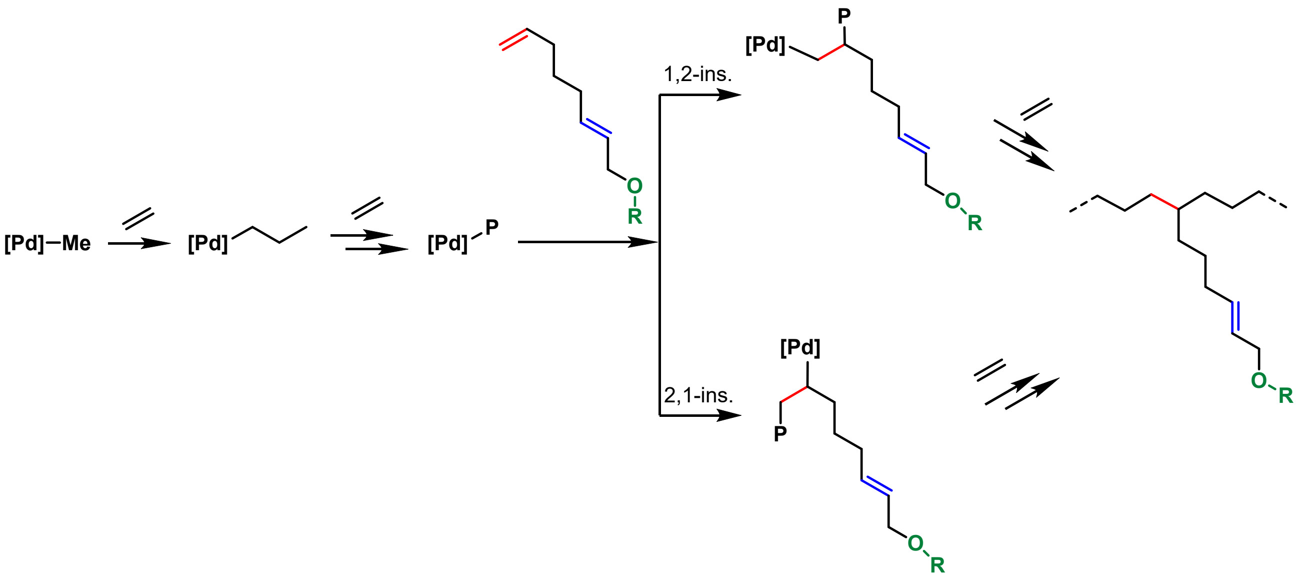

Figure S49. Possible reaction pathways in the copolymerization process.
